# New Asymmetric Gemini Triazole Surfactants with a Polar Triethylene Glycol Fragment: Synthesis and Physico-Chemical Properties

**DOI:** 10.3390/molecules29225420

**Published:** 2024-11-17

**Authors:** Ilshat Bogdanov, Diana Mironova, Elza Sultanova, Vladimir Burilov, Svetlana Solovieva, Igor Antipin

**Affiliations:** 1 Department of Organic and Medicinal Chemistry, Kazan Federal University, Kremlyovskaya Str. 18, 420008 Kazan, Russia; 2 Arbuzov Institute of Organic and Physical Chemistry, FRC Kazan Scientific Center of RAS, Arbuzov Str. 8, 420088 Kazan, Russia

**Keywords:** Gemini surfactant, aggregation property, binding of BSA

## Abstract

The present work is devoted to the synthesis and analysis of the physicochemical properties of new functionalized asymmetric Gemini surfactants. Herein, alkyl- and azide-substituted surfactants with symmetric and asymmetric substituents were synthesized by using the click-reaction method. The critical aggregation concentration values of Gemini surfactants were determined. The binding processes of functionalized Gemini surfactants with bovine serum albumin were evaluated by fluorescence spectroscopy. Also, using the temperature dependences of the binding constants, the mechanism of Gemini surfactants binding with bovine serum albumin was studied. The hydrodynamic diameters of the formed bovine serum albumin/surfactant aggregates were analyzed. Based on electrophoretic light scattering, the ability of the synthesized Gemini surfactants to form associates was analyzed. The possibility of changing the mechanism of interaction in the 15c/bovine serum albumin system was shown. Based on the results obtained using different light scattering techniques and fluorescence spectroscopy, the mechanisms of interaction between bovine serum albumin and surfactants were determined.

## 1. Introduction

Cationic surfactants possess a wide range of physicochemical properties that are important from both fundamental and applied points of view. These compounds have wide application potential, from reaction media for “green” organic transformations and corrosion inhibitors to drug delivery agents or anionic receptors.

Among all cationic surfactants, the so-called Gemini ones have become very popular. Cationic Gemini surfactants consist of two positively charged headgroups connected by a spacer and two lipophilic alkyl moieties. Such structures have better physico-chemical characteristics compared to their monomeric analogs: a lower threshold of critical aggregation concentration, higher solubilizing, wetting and emulsifying abilities etc. [1,2,3,4]. Thus, Gemini surfactants have great potential for use in catalysis, medicine, biochemistry, agriculture, the cosmetics industry, the oil industry, the production of polymer and mesoporous materials, etc.

Recently, cationic Gemini surfactants based on imidazolium derivatives have become the most attractive cationic surfactants. The distinct polarizability of their ring and their high propensity to form hydrogen bonds, π-π-stacking and electrostatic interactions give imidazolium surfactants new and improved properties compared to classical ammonium derivatives [5,6,7]. These amphiphiles have demonstrated an excellent ability to form complexes with protein macromolecules, drugs and nucleic acids. In addition, a number of Gemini imidazolium surfactants exhibit high biological activity against bacterial and fungal infections, as well as cancer cells [8,9,10]. At the moment, these compounds are considered to be promising surfactant molecules for a wide range of applications [6].

One of the relevant aspects of the application of Gemini imidazolium surfactants is the study of their complexing ability with protein molecules. It is known that proteins in the presence of surfactants tend to change their aggregation, morphology and stability, which, in turn, is very important for the medicine, biochemistry, cosmetics and food industries [11,12]. For example, understanding and controlling the interactions between proteins and surfactants will enable new drug delivery systems. Alternatively, a number of studies have shown that geminal imidazole-based surfactants are able to disaggregate amyloid fibrils, so these compounds may become potential candidates for the treatment of Alzheimer’s disease [13].

In recent years, the range of Gemini surfactants has been expanded by the production of asymmetric ones containing alkyl fragments of various lengths [14,15,16,17]. Their properties differ from those of symmetric Gemini surfactants in that they have a lower CAC, high adsorption efficiency, stronger hydrophobic interactions etc.

To the best of our knowledge, there are no examples in the literature of unsymmetrical surfactants containing, on one hand, a lipophilic alkyl substituent, and on the other, a functional polar substituent that imparts the necessary properties to the molecule. Click chemistry, namely the copper-catalyzed cycloaddition reaction CuAAC [18,19,20], opens up enormous prospects for the construction of various asymmetric geminal surfactants. This methodology can also be successfully utilized in the design of new asymmetric geminal surfactants in order to give them new physicochemical properties. In addition, it is worth noting that the triazole ring, due to its ability to form hydrogen bonds and orientational interactions, can also act as an additional binding site for biomolecules.

**Scheme 1:**
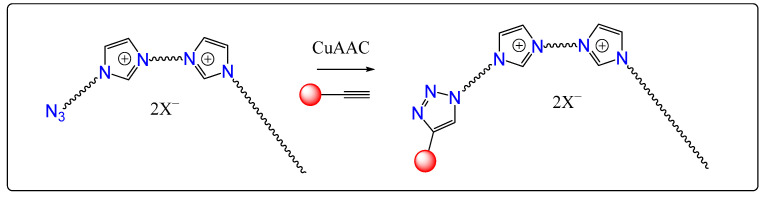
General scheme synthesis of asymmetric Gemini surfactants using CuAAC precursors.

Herein, we present universal approach for the CuAAC synthesis of asymmetric Gemini surfactants using CuAAC precursors–bisimidazolium Gemini molecules with an alkyl fragment on one side and an azide functional group on the other (Scheme 1). The synthesis of asymmetric Gemini azides and triazoles with a polar triethylene glycol fragment, as well as their behavior in water and some of their physicochemical properties, are discussed.

## 2. Results and Discussion

### 2.1. Synthesis of Gemini Surfactants

**Scheme 2:**
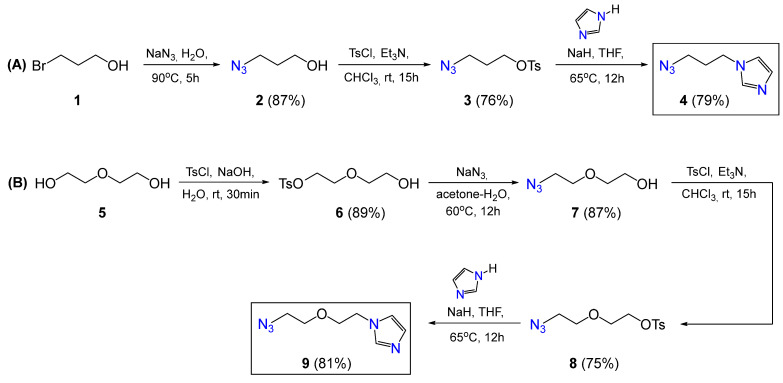
Synthetic pathway for the compounds **4** (**A**) and **9** (**B**).

In the first stage of this work, azide containing imidazole derivatives was synthesized (compounds **4** and **9**). Despite the fact that these compounds are known, the literature methods for their synthesis are inconvenient. Thus, to obtain imidazole **4**, the authors of the work [21] used 3-(1H-imidazole)propan-1-amine, which was then reacted with explosive trifluoromethane or imidazole sulfonyl azides. To obtain compound **9**, the authors of [22] carried out alkylation of imidazole with di(2-chloroethyl)ether to produce imidazole containing a terminal chloride group, which was then subjected to a substitution reaction with sodium azide. This method suffers from a bis-imidazole byproduct. To prepare compounds **4** and **9**, we propose the alkylation of imidazole with tosylate derivatives **3** (Scheme 2A) and **8** (Scheme 2B) in the presence of sodium hydride. Thus, compounds **4** and **9** were obtained in 79% and 81% yields, respectively.

To ensure the covalent binding of two imidazolium fragments, it was decided to obtain a series of imidazolium salts containing *n*-butyl, *n*-octyl and *n*-tetradecyl fragments at the first nitrogen atom, as well as a propylene linker with a terminal bromide group at the third nitrogen atom. For this, the quaternization of alkylimidazoles with a large excess of 1,3-di bromopropane (10–12 eq) in acetone at 50 °C can be used [23,24,25].

**Scheme 3:**
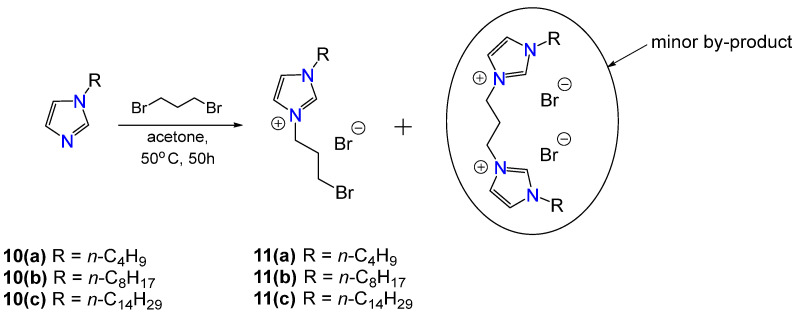
Synthesis of bisimidazolium derivatives **11a**–**c**.

Thus, *N*-alkylimidazoles **10a**–**c** reacted with a twelvefold excess of 1,3-dibromopropane (Scheme 3). Syntheses were carried out at high dilution in acetone at 50 °C. Using GCMS, it was found that complete conversion of the starting substrates was achieved within 50 h. Despite the large excess of 1,3-dibromopropane, the formation of symmetric bisimidazolium derivatives as impurities was found. Several standard methods were used to purify target compounds, and none of them proved effective.

**Scheme 4:**
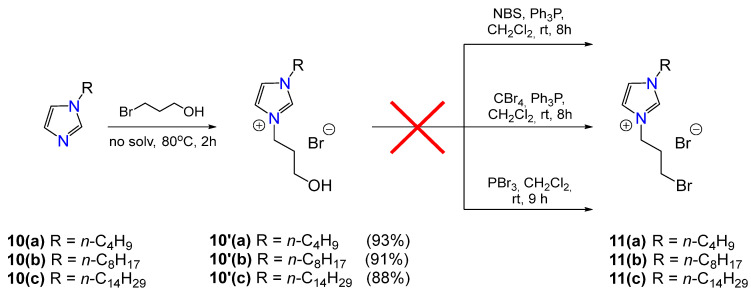
Synthetic pathway of the quaternization of *N*-alkylimidazoles **10a**–**c** for obtaining compounds **11a**–**c**.

Next, an alternative method for obtaining compounds **11a**–**c** was proposed. It consisted of the quaternization of *N*-alkylimidazoles **10a**–**c** with a 3-bromopropanol-1 followed by the introduction of the obtained imidazolium salts **10′a**–**c** to the bromination stage (Scheme 4). Compounds **10′a**–**c** were obtained using the method described in [26] and were involved in a reaction with three standard bromination reagents: NBS/PPh_3_, CBr_4_/PPh_3_ and PBr_3_. However, none of them led us to obtain compounds **11a**–**c** in pure form.

**Scheme 5:**
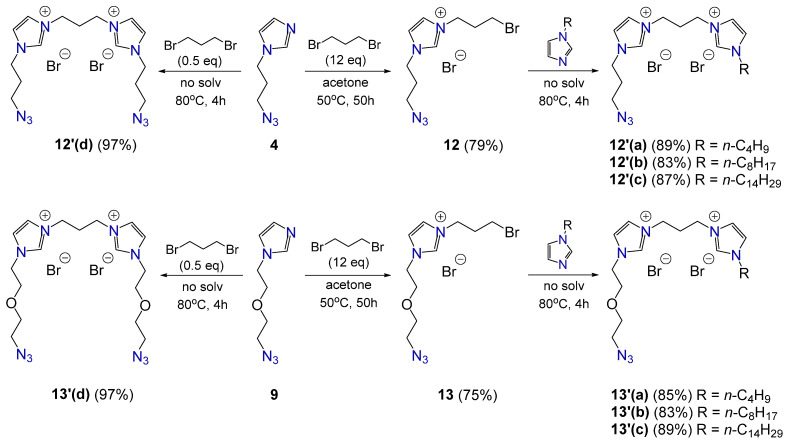
Synthetic pathway for the compounds **12′a**–**c** and **13′a**–**c**.

Therefore, the synthetic strategy was modified. Instead of alkylimidazoles **10a**–**c**, the previously obtained azide imidazole derivatives **4** and **9** were introduced into the quaternization reaction. The syntheses were carried out similarly to those in the procedure of compounds **11a**–**c** (Scheme 5). In these cases, the formation of minor symmetrical bis-imidazolium derivatives also occurred. However, compounds **12**–**13** were successfully purified by SiO_2_ column chromatography (eluent: acetone/methanol = 10/1).

New compounds were proven by a complex of physical methods. Thus, in the ^1^H NMR spectrum of compound **12**, the appearance of triplets of -CH_2_N_3_ and -CH_2_Br protons is observed at δ 3.44 ppm (*J* = 6.6 Hz) and δ 3.54 ppm (*J* = 6.5 Hz), respectively. The protons of the methylene groups connected with the imidazolium ring appear at δ 4.24 ppm (*J* = 7.0 Hz) and δ 4.29 ppm (*J* = 6.3 Hz) in the form of triplets. The acidic imidazolium proton at the C2 atom appears as a singlet at δ 9.29 ppm, and at δ 7.85 ppm, there is an imposition of signals from the C4 and C5 imidazole ring protons. In the ^13^C NMR spectrum, signals from the carbon atoms of the imidazolium ring appear at δ 122 ppm and δ 136 ppm. In the IR spectra of the obtained compounds **12**–**13**, the most characteristic is the presence of an intense absorption band at 2100 cm^−1^, which corresponds to asymmetric stretching vibration of the azide group. In the high-resolution (HR) ESI mass spectra of compounds **12**–**13**, peaks of quasi-molecular ions [M-Br]^+^ were found.

Next, compounds **12**–**13** were reacted with an equimolar amount of *N*-alkylimidazoles **10a**–**c** (Scheme 5). Syntheses were carried out without solvent at a temperature of 80 °C. Four hours after the start of stirring, an increase in the viscosity of the reaction mixture was observed. Using GCMS, complete conversion of the starting *N*-alkylimidazoles was established. After isolation, a series of unsymmetrical bisimidazolium salts (compounds **12′a**–**c** and **13′a**–**c**) containing an alkyl moiety on one imidazolium head group and a propyl/oxyethyl azide moiety on the other were obtained in high yields. Also, symmetrical azide-containing bisimidazolium salts **12′d** and **13′d** were obtained in almost quantitative yields (Scheme 5). For this, 1 eq of 1,3-dibromopropane with 2 eq of imidazoles **4** or **9** was stirred at 80 °C.

The structure of the compounds is fully proven and confirmed by a complex of physical methods. In the ^1^H NMR spectrum of compound **12′a**, the most characteristic is the shift in the signal from the protons of the brommethylene fragment to the downfield region (∆δ 1.27 ppm), as well as the signal shift in the linker’s methylene protons (∆δ 0.07 ppm). Also, the appearance of new signals for the protons of the alkyl substituent is observed at δ 1.79 ppm, δ 1.26 ppm and δ 0.90 ppm (Figure S1). For the symmetric azide derivative **12′d** in the ^1^H NMR spectrum, there is an overlap of signals of methylene protons associated with imidazolium rings at δ 4.20–4.30 ppm, and for compound **13′d,** due to the presence of an electronegative oxygen atom, protons of methylene fragments associated with imidazolium nuclei are clearly resolved and appear as triplets at δ 4.29 ppm (*J* = 6.6 Hz) and δ 4.41 ppm (*J* = 6.7 Hz). In the HR ESI mass spectra of bisimidazolium azide derivatives, peaks [M-2Br]^2+^ of doubly charged quasi-molecular ions were found in all cases.

**Scheme 6:**
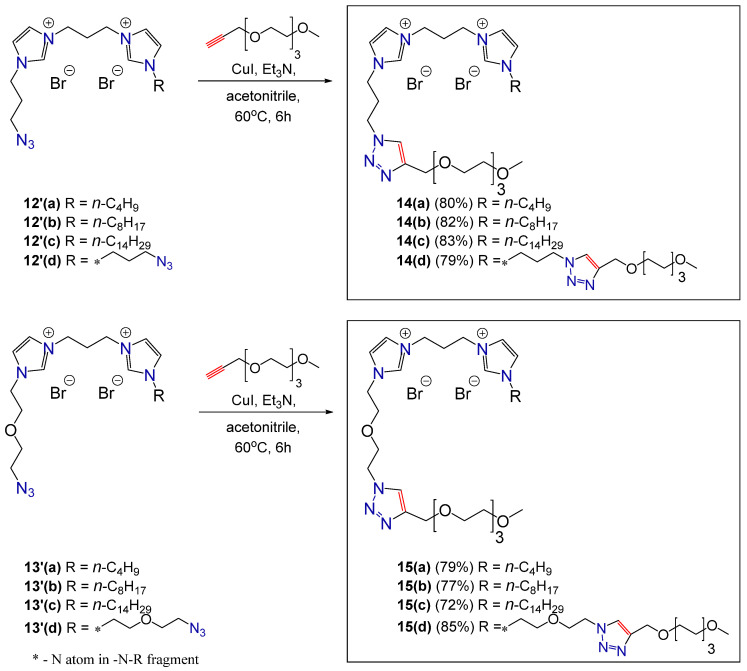
Synthetic pathway for the compounds **14′a**–**c** and **15′a**–**c**.

Finally, compounds **12′a**–**d** and **13′a**–**d** were introduced into the azide-alkyne cycloaddition reaction with triethylene glycol propargyl methyl ether (Scheme 6). The synthesis took place in acetonitrile with a СuI-NEt_3_ catalytic system. To purify target compounds from copper, aqueous solutions of compounds were passed through Amberlite IRA-67. After washing the aqueous solution with diethyl ether and water evaporation, a series of triazole-bis-imidazolium derivatives, **14a**–**d** and **15a**–**d**, were obtained in 72–85% yield.

In the ^1^H NMR spectrum of compound **14a**, a singlet of the methoxyl protons appears at δ 3.21 ppm, as well as multiplets from the protons of the oxyethyl fragments at δ 3.39–3.43 ppm and δ 3.47–3.58 ppm. The signal of the methylene proton close to the C4 atom of the triazole ring appears as a singlet at δ 4.51 ppm, and the proton at the C5 triazole atom at δ 8.19 ppm. The methylene protons near the triazole ring fall into the deshielding zone and undergo a downfield shift at ∆δ 1.01 ppm and ∆δ 0.36 ppm (Figure S2). In the ^13^C NMR spectra, the most characteristic is the appearance of signals at δ 124 ppm and δ 144 ppm, corresponding to the signals of the C5 and C4 atoms of the triazole, respectively. In the IR spectra of the obtained compounds, the disappearance of the absorption band of asymmetric stretching vibrations of the azide group at 2100 cm^−1^ is observed. The HR ESI mass spectra of compounds **14a**–**d** and **15a**–**d** contain peaks [M-2Br]^2+^ of doubly charged quasi-molecular ions.

### 2.2. CAC Studies of Gemini Surfactant

The ability of imidazolium-based Gemini surfactants to form aggregates in aqueous solutions and their critical aggregation concentrations (CAC) were determined. This is a common method based on well-known fluorometric measurement of the ratio of intensities of the first (373 nm) and third (383 nm) maxima in the pyrene emission spectrum, which are extremely sensitive to changes in the polarity of the medium during the solubilization of pyrene in the hydrophobic layer of micelles or vesicles (Figure S21). Since the asymmetric surfactants **14d** and **15d** have short hydrophobic substituents and the symmetric surfactants **14a** and **15a** have no amphiphilic properties, it was not possible to determine the CAC value for them. In the case of compounds **15b**, **15c**, **14b** and **14c**, the CAC values were evaluated and are presented in Table 1. The surfactants with tetradecyl substituents **15c** and **14c** have close CAC values and aggregate at lower concentration compared to surfactants **15b** and **14b** containing octyl substituents and a single-chain imidazolium surfactant with tetradecyl alkyl substituents. Thus, the aggregation of surfactants **15b** and **14b** at higher concentrations is due to the fact that the octyl hydrophobic substituents are not sufficient for hydrophilic–lipophilic balance to be observed in the compound. Data on the hydrodynamic diameter and zeta potential values for surfactants at a concentration above the CAC for surfactants (Table 1) were obtained.

### 2.3. Fluorescence Spectra of BSA with Gemini Surfactant

The possibility of imidazole-based ionic liquids binding to various protein molecules has been shown previously [27,28,29]. It has been shown that surfactant ionic liquids can bind to protein molecules at low concentrations and exhibit different types of intermolecular interactions depending on the surfactant structure [30,31,32].

**Figure 1:**
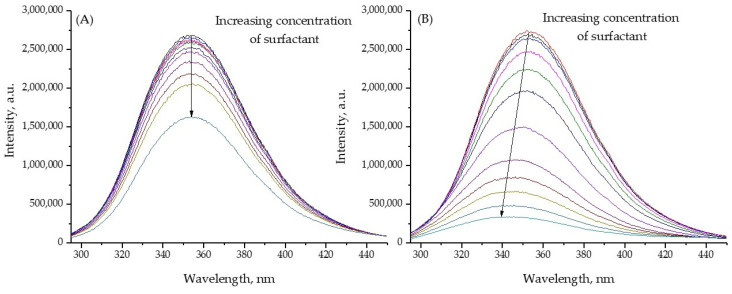
Emission spectra of BSA system in absence and presence of various concentrations of **15a** (**A**) and **15c** (**B**) in 25 mM Tris-HCl buffer solution with pH 7.4, [BSA] 10 μM, [**15a**] = [**15c**] = 0–700 μM.

Fluorescence spectroscopy is a well-recognized method for assessing changes in the microenvironment in the vicinity of the chromophore. Thus, there are two tryptophan residues (Trp 134 and Trp 213) in the BSA structure, located in domain IB and in a hydrophobic pocket in domain IIA [33]. In Figure 1 (all emission spectra of BSA in the presence of **14a**–**d** and **15a**–**d** are presented in Supplementary Materials), the fluorescence spectra of BSA with surfactants are presented.

For all investigated surfactants, quenching of the fluorescence emission of BSA from the concentration of the added surfactant (0–700 μM) is observed. However, in the case of **14c** and **15c**, it is found that the fluorescence intensity decreases with a slight blue shift (~11 nm). The blue shift in the emission maximum at 11 nm indicates that in the presence of the more lipophilic **14c** and **15c**, the Trp residues have a more hydrophobic environment compared to their native state—the hydrophobic regions of the surfactant shield the Trp residues from the polar aqueous environment [34]. Alternatively, the change in the environment of tryptophan residues could be the result of the unfolding of BSA and the changes in the conformation of the BSA molecules upon the adsorption of **14c** and **15c** [35,36].

The Stern–Volmer Equation (1) was used to quantify the quenching efficiency of surfactants binding to BSA in the vicinity of the fluorophore

(1) $$\frac{F_{0}}{F}=1+K_{SV}[Q]$$

where $F_{0}$ and F are the fluorescence intensities of BSA in the absence and presence of quenchers (surfactants), [Q] is the quencher concentration, and $K_{SV}$ is the Stern–Volmer quenching constant. The calculated values for all surfactants are presented in Table 2. The obtained values of $K_{SV}$ are similar $K_{SV}$, with the exception of **15c**.

To quantify the binding parameters of surfactants with BSA, the following equation can be used:

(2) $$\log\frac{F_{0}-F}{F}=\log K_{b}+n\log[Q]$$

where n is the number of binding sites and $K_{b}$ is the binding constant. The obtained binding parameters are presented in Table 1. The obtained values of the binding constants of the surfactants with BSA at 298 K are small, because the binding interval of BSA with ligands are at the level of 10^5^–10^9^ M^−1^ [37], which may indicate the absence of the specific binding of surfactants with BSA.

The complexation between ligand and protein occurs due to various intermolecular interactions, such as hydrogen bonding, van der Waals, electrostatic and hydrophobic forces. The temperature dependence of the binding constant according to the Van‘t Hoff equation allows us to estimate the thermodynamic parameters of BSA binding to surfactants:

(3) ΔG=ΔH−TΔS

(4) $$\ln K=-\frac{\Delta H}{RT}+\frac{\Delta S}{R}$$

In Table 2 the thermodynamic binding parameters of the surfactant with BSA are presented. The binding enthalpies and entropies of all surfactants with BSA are positive, with the exception of **15c**.

The increase in alkyl radical length in the series **14a**–**14c** leads to an increase in the binding enthalpy of the Gemini surfactant to BSA. The endothermic enthalpy and entropy of binding indicate the hydrophobic nature of the binding between the Gemini surfactant and BSA [38]. In the case of **14d**, a lower binding enthalpy relative to **14a** is observed, which may be due to the better solvation of **14d** in water as a result of the additional triazole group in the structure of the Gemini surfactant. However, for Gemini surfactants in the series **15a**–**15d**, no dependence of the binding enthalpy on the length of the alkyl substituent was observed. This may be due to the content of the oxyethylene moiety, which allows the Gemini surfactants to better solvate in water, causing the binding enthalpy to become less endothermic. However, for the **15b**/BSA system, a strong increase in the binding enthalpy was found compared to **14b**, possibly due to a larger entropic contribution associated with the rearrangement of the water molecules that surrounded the protein surface [39]. In the case of **15c**, the exothermic enthalpy and endothermic entropy are obtained. They indicate the electrostatic nature of the behavior of the surfactant and protein molecule [40]. Apparently, the long alkyl substituents in the case of **15c** have a special conformational arrangement, resulting in the screening of other active fragments (triazoles). However, such a phenomenon is not observed for **14c**, since its structure lacks an oxyethyl moiety, which presumably also participates in electrostatic interactions together with imidazolium rings. Also, the small enthalpy of the binding of **15c** to BSA may indicate a key role of the oxyethyl moiety.

The binding of **14c** to BSA in different pH (pH = 6 and pH-9) conditions was studied. The surfactant **14c** was chosen since it has the highest binding constant with BSA at 298.15 K. The obtained results of the kinetic parameters of binding at different pH values are summarized in Table 2. At pH = 6, a decrease in the binding constant of **14c** to BSA was observed. This can be related to the decrease the negative charge on the BSA surface, since the isoelectric point of BSA ranges from pH 5.1 to 5.5 [41]. At pH = 9, an increase in the binding of **14c** to BSA is observed.

### 2.4. Hydrodynamic Diameter and Zeta-Potential of BSA in Presence of Gemini Surfactant

The aggregate formation between BSA with surfactants was measured using the dynamic and electrophoretic light scattering techniques (Figure S24). In Table 3 the hydrodynamic diameter values and polydispersity index surfactant/BSA systems are presented. At concentrations corresponding to the binary mixture (surfactant/BSA), the studied surfactants do not form associations. For all investigated surfactant/BSA systems, there is practically no change in diameter relative to pure BSA. This fact agrees well with the obtained data on the thermodynamics of the surfactant/protein interaction. According to these data, the mechanism of hydrophobic interaction is determined for all surfactants; in such an interaction, the ligands usually penetrate into the hydrophobic cavities of the protein. During this penetration, the hydrodynamic diameter does not change significantly [42]. In the case of **15c**, strong aggregation in the BSA solution is observed, in contrast to the other surfactants. This result also agrees well with the data on the thermodynamics of binding to the protein molecule. According to these data, **15c** has an expressed electrostatic nature of interaction, which apparently allows **15c** to strongly aggregate around BSA. In Table 3, the zeta potential values for all studied surfactants are presented. The obtained values for the zeta potential of the surfactants are rather low, which may indicate the tendency of these systems to aggregate. Indeed, for **15a**, the PDI value is better than for **15c**. For all studied systems, the PDI is worse than for **15a**, and according to the measured zeta potential, all of the studied systems are prone to aggregation. However, only for **15c** is an increase in the hydrodynamic diameter observed. Also, the analysis of thermodynamic parameters indicates the electrostatic nature of the interaction of **15c** with BSA, which may additionally indicate its ability to aggregate. This type of aggregation is similar to the behavior of ionic liquids with BSA, where strong aggregation upon interaction is also observed [43].

## 3. Materials and Methods

### 3.1. Experimental Sections

Commercially available reagents from the catalogs of Acros Organics, Macklin, Sigma Aldrich and Alpha Aesar were used. Solvents were preliminarily purified using standard methods. THF was additionally boiled and distilled over sodium. Acetonitrile was additionally distilled by phosphorus pentoxide.

### 3.2. Sample Preparation

All measurements (fluorescence spectroscopy, dynamic and electrophoretic light scattering) were performed in buffer solution (Tris buffer, phosphate buffer). BSA solutions were prepared by dissolving the BSA powder in buffer through mild stirring at room temperature.

### 3.3. General Procedure for Synthesis of Gemini Surfactants

The purity of substances was controlled by TLC on Merck UV 254 plates with manifestation in UV light from a VL-6.LC lamp. NMR experiments were performed at room temperature on a Bruker Avance 400 Nanobay spectrometer. The IR spectra of the obtained compounds were recorded on a Bruker Vector-22 FT-IR spectrometer in the wave number range of 400–4000 cm^−1^ in KBr pellets. HRMS-ESI mass spectra were obtained on an Agilent iFunnel 6550 LC/Q-TOF mass spectrometer. Gas chromatography–mass spectrometry was performed on a GCMS-QP2010 Ultra gas chromatography–mass spectrometer (Shimadzu, Kyoto, Japan) equipped with an HP-5MS column (the internal diameter was 0.32 mm, and the length was 30 m). The parameters were as follows: helium of 99.995% purity was the carrier gas, the temperature of the injector was 250 °C, the flow rate through the column was 2 mL/min, and the thermostat temperature program was a gradient temperature increase from 70 to 250 °C with a step of 10 °C/min. The range of the scanned masses was *m*/*z* 35–400. The internal standard method using dodecane was used for the quantitative analysis.

3-azidopropanol-1 **2** [44], 3-azidopropyl-4-methylbenzenesulfonate **3** [12], 2-(2-hydroxyethoxy)ethyl-4-methylbenzenesulfonate **6** [45], 2-(2-azidoethoxy)ethanol **7** [46], 2-(2-azidoethoxy)ethyl-4-methylbenzenesulfonate **8** [47], 1-butyl-1H-imidazole **10a**, 1-octyl-1H-imidazole **10b**, 1-tetradecyl-1H-imidazole **10c** [48] and triethylene glycol methyl propargyl ether [49] were obtained using literary methods.

General procedure for preparation of N-substituted imidazoles **4** and **9**.

A total of 19.1 mmol of sodium hydride was added to a solution of 14.7 mmol of imidazole in 50 mL of THF in the cold. After its addition, the mixture was stirred for 15 min in an ice bath. Then, 16.2 mmol of compound **3** or **8** was added to the finished suspension and stirred at 65 °C for 18 h. After this, the THF was distilled off on a rotor. The resulting dry residue was dissolved in 80 mL of saturated sodium chloride solution. The aqueous phase was extracted with chloroform (3 × 80 mL), and the combined organic layers were dried over magnesium sulfate and evaporated on a rotary evaporator. The target product, compound **4** or **9**, was purified by flash chromatography (ethyl acetate–methanol).

General procedure for preparation of 3-bromopropyl-N-alkyl imidazolium salts containing azide groups.

A total of 60 mmol of 1,3-dibromopropane was added to 5 mmol of compounds **4** or **9**. The resulting mixture was dissolved in 50 mL of acetone and stirred at 50 °C for 50 h. After this, the solvent and excess dibromopropane were evaporated on a rotary evaporator. The target product was purified by column chromatography (acetone/methanol = 10/1).

3-(3-azidopropyl)-1-(3-bromopropyl)-1H-imidazolium bromide (**12**)**.** Yield 1.39 g (79%). NMR ^1^H (400 MHz, DMSO-d_6_, 25 °C) δH, ppm.: 9.29 (s, 1H, ImH), 7.85 (br s, 2H, ImH), 4.29 (t, *J* = 6.9 Hz, 2H, CH_2_Im), 4.24 (t, *J* = 7.0 Hz, 2H, CH_2_Im), 3.54 (t, *J* = 6.5 Hz, 2H, CH_2_Br), 3.44 (t, *J* = 6.6 Hz, 2H, CH_2_N_3_), 2.42–2.33 (m, 2H, CH_2_), 2.11–2.03 (m, 2H, CH_2_). NMR ^13^C{^1^H} (100.9 MHz, DMSO-d_6_, 25 °C) δC, ppm.: 136.6, 122.6, 122.5, 47.6, 47.6, 46.5, 32.1, 30.6, 28.6. IR (KBr) ν_max_, cm^−1^: ν 3134 m (С_Ar_-H), ν_as_ 2103 s (-N_3_), ν 1564 s (C=C), ν 1455 m (C-N), ν 639 m (C-Br). HRMS-ESI: found to be *m*/*z* 272.0506 [M-Br]^+^; calculated for C_9_H_15_BrN_5_^+^ 272.0505.

3-(2-(2-azidoethoxy)ethyl)-1-(3-bromopropyl)-1H-imidazolium bromide **(13**)**.** Yield 1.44 g (75%). NMR ^1^H (400 MHz, CD_3_CN-d_3_, 25 °C) δH, ppm.: 9.43 (s, 1H, ImH), 7.66 (s, 1H, ImH), 7.66 (s, 1H, ImH), 4.44 (t, *J* = 4.8 Hz, 2H, CH_2_Im), 4.40 (t, *J* = 7.0 Hz, 2H, CH_2_Im), 3.85 (t, *J* = 5.0 Hz, 2H, CH_2_O), 3.65 (t, *J* = 4.7 Hz, 2H, CH_2_O), 3.49 (t, *J* = 6.5 Hz, 2H, CH_2_Br), 3.37 (t, *J* = 5.0 Hz, 2H, CH_2_N_3_), 2.47–2.38 (m, 2H, CH_2_). NMR ^13^C{^1^H} (100.9 MHz, CD_3_CN-d_3_, 25 °C) δC, ppm.: 137.6, 123.8, 123.1, 70.4, 69.1, 51.2, 50.2, 48.7, 33.2, 30.5. IR (KBr) ν_max_, cm^−1^: ν 3144 m (С_Ar_-H), ν_as_ 2113 s (-N_3_), ν 1564 s (C=C), ν 1445 m (C-N), ν 641 m (C-Br). HRMS-ESI: found to be *m*/*z* 302.0611 [M-Br]^+^; calculated for C_10_H_17_BrN_5_O^+^ 302.0611.

General procedure for preparation of bis-imidazolium derivatives **12′** and **13′** with azide functional groups.

To obtain unsymmetrical salts, an equimolar amount of compound **12** or **13** was added to 1.5 mmol of *N*-alkylimidazole **10a** or **10b** or **10c**. The mixture was stirred at 80 °C for 4 h, and was then dissolved in water (10 mL) and washed with diethyl ether (2 × 15 mL). The water was evaporated to obtain target compounds as yellow oils. To obtain a symmetrical salt, 0.5 mmol of 1,3-dibromopropane was added to 0.5 mmol of compound 4 or 9. The synthesis procedure was similar to that for the unsymmetrical salts.

3-(3-azidopropyl)-1-(3-(1-butyl-1H-imidazol-3-yl)propyl)-1H-imidazolium dibromide (**12′a**). Yield 0.42 g (89%). NMR ^1^H (400 MHz, DMSO-d_6_, 25 °C) δH, ppm.: 9.43–9.38 (m, 2H, ImH), 7.90–7.85 (m, 4H, ImH), 4.31–4.23 (m, 6H, CH_2_Im), 4.19 (t, *J* = 7.2 Hz, 2H, CH_2_Im), 3.46 (t, *J* = 6.6 Hz, 2H, CH_2_N_3_), 2.48–2.39 (m, 2H, CH_2_), 2.14–2.04 (m, 2H, CH_2_), 1.83–1.75 (m, 2H, CH_2_), 1.32–1.22 (m, 2H, CH_2_), 0.90 (t, *J* = 7.4 Hz, 3H, CH_3_). NMR ^13^C{^1^H} (100.9 MHz, DMSO-d_6_, 25 °C) δC, ppm.: 136.6, 136.3, 122.5, 122.4, 122.3, 48.6, 47.6, 46.5, 45.81, 45.8, 31.2, 29.5, 28.6, 18.8, 13.1. IR (KBr) ν_max_, cm^−1^: ν 3070 m (С_Ar_-H), ν_as_ 2936 m (СH_2_), ν_as_ 2101 s (-N_3_), ν 1644 m (С=N), ν 1564 s (C=C), ν 1458 m (С-N). HRMS-ESI: found to be *m*/*z* 158.6158 [M-2Br]^2+^; calculated for C_16_H_27_N_7_^2+^ 158.6158.

3-(3-azidopropyl)-1-(3-(1-octyl-1H-imidazol-3-yl)propyl)-1H-imidazolium dibromide (**12′b**). Yield 0.66 g (83%). NMR ^1^H (400 MHz, DMSO-d_6_, 25 °C) δH, ppm.: 9.42 (br s, 2H, ImH), 7.93–7.85 (m, 4H, ImH), 4.33–4.24 (m, 6H, CH_2_Im), 4.19 (t, *J* = 7.2 Hz, 2H, CH_2_Im), 3.47 (t, *J* = 6.6 Hz, 2H, CH_2_N_3_), 2.49–2.41 (m, 2H, CH_2_), 2.15–2.03 (m, 2H, CH_2_), 1.85–1.76 (m, 2H, CH_2_), 1.31–1.21 (m, 10H, CH_2_), 0.86 (t, *J* = 6.4 Hz, 3H, CH_3_). NMR ^13^C{^1^H} (100.9 MHz, DMSO-d_6_, 25 °C) δC, ppm.: 136.6, 136.3, 122.6, 122.6, 122.5, 122.4, 48.9, 47.6, 46.6, 45.9, 45.9, 31.2, 29.5, 29.3, 28.6, 28.5, 28.4, 25.6, 22.1, 14.0. IR (KBr) ν_max_, cm^−1^: ν 3074 m (С_Ar_-H), ν_as_ 2929 m (СH_2_), ν_as_ 2100 s (-N_3_), ν 1630 m (С=N), ν 1565 s (C=C), ν 1457 m (С-N). HRMS-ESI: found to be *m*/*z* 186.6471 [M-2Br]^2+^; calculated for C_20_H_35_N_7_^2+^ 186.6471.

3-(3-azidopropyl)-1-(3-(1-tetradecyl-1H-imidazol-3-yl)propyl)-1H-imidazolium dibromide (**12′c**). Yield 0.80 g (87%). NMR ^1^H (400 MHz, DMSO-d_6_, 25 °C) δH, ppm.: 9.41 (br s, 2H, ImH), 7.91–7.85 (m, 4H, ImH), 4.31–4.22 (m, 6H, CH_2_Im), 4.17 (t, *J* = 7.3 Hz, 2H, CH_2_Im), 3.46 (t, *J* = 6.6 Hz, 2H, CH_2_N_3_), 2.48–2.36 (m, 2H, CH_2_), 2.13–2.04 (m, 2H, CH_2_), 1.84–1.74 (m, 2H, CH_2_), 1.34–1.18 (m, 22H, CH_2_), 0.84 (t, *J* = 6.7 Hz, 3H, CH_3_). NMR ^13^C{^1^H} (100.9 MHz, DMSO-d_6_, 25 °C) δC, ppm.: 136.6, 136.3, 122.5, 122.5, 122.5, 122.4, 64.9, 48.8, 47.6, 46.5, 45.8, 45.8, 31.3, 29.4, 29.3, 29.1, 29.0, 29.0, 28.9, 28.7, 28.6, 28.4, 25.6, 22.1, 15.1, 13.9. IR (KBr) ν_max_, cm^−1^: ν 3052 m (С_Ar_-H), ν_as_ 2957 m (СH_2_), ν_as_ 2101 s (-N_3_), ν 1628 m (С=N), ν 1561 m (C=C), ν 1451 m (С-N). HRMS-ESI: found to be *m*/*z* 228.6942 [M-2Br]^2+^; calculated for C_26_H_47_N_7_^2+^ 228.6941.

3,3′-(propane-1,3-diyl)bis(1-(3-azidopropyl)-1H-imidazol-3-ium) dibromide (**12′d**). Yield 0.24 g (97%). NMR ^1^H (400 MHz, DMSO-d_6_, 25 °C) δH, ppm.: 9.29 (s, 2H, ImH), 7.85 (br s, 2H, ImH), 7.84 (br s, 2H, ImH), 4.30–4.20 (m, 8H, CH_2_Im), 3.45 (t, *J* = 6.6 Hz, 4H, CH_2_N_3_), 2.46–2.37 (m, 2H, CH_2_), 2.13–2.03 (m, 4H, CH_2_). NMR ^13^C{^1^H} (100.9 MHz, DMSO-d_6_, 25 °C) δC, ppm.: 136.5, 122.6, 122.5, 47.6, 46.6, 45.9, 29.4, 28.6. IR (KBr) ν_max_, cm^−1^: ν 3079 m (С_Ar_-H), ν_as_ 2942 m (СH_2_), ν_as_ 2101 s (-N_3_), ν 1621 m (С=N), ν 1545 s (C=C), ν 1453 m (С-N). HRMS-ESI: found to be *m*/*z* 172.1089 [M-2Br]^2+^; calculated for C_15_H_24_N_10_^2+^ 172.1087.

1-(2-(2-azidoethoxy)ethyl)-3-(3-(1-butyl-1H-imidazol-3-yl)propyl)-1H-imidazolium dibromide (**13′a**). Yield 0.64 g (85%). NMR ^1^H (400 MHz, DMSO-d_6_, 25 °C) δH, ppm.: 9.27 (s, 1H, ImH), 9.24 (s, 1H, ImH), 7.84 (br s, 2H, ImH), 7.81 (br s, 2H, ImH), 4.39 (t, *J* = 4.5 Hz, 2H, CH_2_Im), 4.27–4.22 (m, 4H, CH_2_Im), 4.18 (t, *J* = 7.2 Hz, 2H, CH_2_Im), 3.83 (t, *J* = 4.8 Hz, 2H, CH_2_O), 3.64 (t, *J* = 4.6 Hz, 2H, CH_2_O), 3.40 (t, *J* = 4.8 Hz, 2H, CH_2_N_3_), 2.46–2.36 (m, 2H, CH_2_), 1.85–1.73 (m, 2H, CH_2_), 1.34–1.22 (m, 2H, CH_2_), 0.91 (t, *J* = 7.4 Hz, 3H, CH_3_). NMR ^13^C{^1^H} (100.9 MHz, DMSO-d_6_, 25 °C) δC, ppm.: 136.7, 136.3, 122.8, 122.5, 122.3, 122.2, 69.2, 67.8, 49.8, 48.8, 48.6, 45.8, 31.2, 29.6, 18.8, 13.3. IR (KBr) ν_max_, cm^−1^: ν 3061 m (С_Ar_-H), ν_as_ 2952 cр. (СH_2_), ν_as_ 2110 s (-N_3_), ν 1629 m (С=N), ν 1564 s (C=C), ν 1448 m (С-N), ν_as_ 1072 m (C-O-C). HRMS-ESI: found to be *m*/*z* 173.6212 [M-2Br]^2+^; calculated for C_17_H_29_N_7_O^2+^ 173.6211.

1-(2-(2-azidoethoxy)ethyl)-3-(3-(1-octyl-1H-imidazol-3-yl)propyl)-1H-imidazolium dibromide (**13′b**). Yield 0.70 g (83%). NMR ^1^H (400 MHz, DMSO-d_6_, 25 °C) δH, ppm.: 9.33 (s, 1H, ImH), 9.30 (s, 1H, ImH), 7.91–7.78 (m, 4H, ImH), 4.40 (br s, 2H, CH_2_Im), 4.26 (br s, 4H, CH_2_Im), 4.17 (br s, 2H, CH_2_Im), 3.84 (br s, 2H, CH_2_O), 3.64 (br s, 2H, CH_2_O), 3.40 (br s, 2H, CH_2_N_3_), 2.41 (br s, 2H, CH_2_), 1.80 (br s, 2H, CH_2_), 1.25 (br s, 10H, CH_2_), 0.85 (br s, 3H, CH_3_). NMR ^13^C{^1^H} (100.9 MHz, DMSO-d_6_, 25 °C) δC, ppm.: 136.7, 136.3, 122.8, 122.5, 122.3, 122.21, 69.2, 67.8, 49.8, 48.9, 48.8, 45.8, 31.1, 29.6, 29.6, 28.5, 28.3, 25.5, 22.0, 13.92. IR (KBr) ν_max_, cm^−1^: ν 3075 m (С_Ar_-H), ν_as_ 2928 m (СH_2_), ν_as_ 2110 s (-N_3_), ν 1626 m (С=N), ν 1566 s (C=C), ν 1456 m (С-N), ν_as_ 1027 m (C-O-C). HRMS-ESI: found to be *m*/*z* 201.6525 [M-2Br]^2+^; calculated for C_21_H_37_N_7_O^2+^ 201.6524.

1-(2-(2-azidoethoxy)ethyl)-3-(3-(1-tetradecyl-1H-imidazol-3-yl)propyl)-1H-imidazolium dibromide (**13′c**). Yield 0.86 g (89%). NMR ^1^H (400 MHz, DMSO-d_6_, 25 °C) δH, ppm.: 9.41 (s, 1H, ImH), 9.37 (s, 1H, ImH), 7.91–7.85 (m, 3H, ImH), 7.82 (s, 1H, ImH), 4.41 (t, *J* = 4.6 Hz, 2H, CH_2_Im), 4.32–4.25 (m, 4H, CH_2_Im), 4.17 (t, *J* = 7.2 Hz, 2H, CH_2_Im), 3.85 (t, *J* = 4.7 Hz, 2H, CH_2_O), 3.65 (t, *J* = 4.6 Hz, 2H, CH_2_O), 3.40 (t, *J* = 4.8 Hz, 2H, CH_2_N_3_), 2.48–2.37 (m, 2H, CH_2_), 1.86–1.74 (m, 2H, CH_2_), 1.35–1.10 (m, 22H, CH_2_), 0.84 (t, *J* = 6.4 Hz, 3H, CH_3_). NMR ^13^C{^1^H} (100.9 MHz, DMSO-d_6_, 25 °C) δC, ppm.: 137.8, 137.4, 123.8, 123.4, 123.2, 123.2, 70.5, 69.0, 51.3, 50.5, 50.5, 47.2, 47.1, 32.6, 30.8, 30.5, 30.3, 30.3, 30.3, 30.2, 30.1, 30.0, 29.6, 26.7 23.3, 14.3. IR (KBr) ν_max_, cm^−1^: ν 3069 m (С_Ar_-H), ν_as_ 2946 m (СH_2_), ν_as_ 2104 s (-N_3_), ν 1628 m (С=N), ν 1559 s (C=C), ν 1442 s (С-N), ν_as_ 1079 m (C-O-C). HRMS-ESI: found to be *m*/*z* 243.6996 [M-2Br]^2+^; calculated for C_27_H_49_N_7_O^2+^ 243.6994.

3,3′-(propane-1,3-diyl)bis(1-(2-(2-azidoethoxy)ethyl)-1H-imidazol-3-ium) dibromide (**13′d**). Yield 0.27 g (97%). NMR ^1^H (400 MHz, DMSO-d_6_, 25 °C) δH, ppm.: 9.41 (br d, 2H, ImH), 7.95–7.88 (m, 2H, ImH), 7.84 (br s, 2H, ImH), 4.41 (t, *J* = 4.7 Hz, 4H, ImCH_2_), 4.29 (t, *J* = 6.6 Hz, 4H, CH_2_Im), 3.85 (t, *J* = 4.9 Hz, 4H, CH_2_O), 3.64 (t, *J* = 4.6 Hz, 4H, CH_2_O), 3.40 (t, *J* = 4.9 Hz, 4H, CH_2_N_3_), 2.46–2.38 (m, 2H, CH_2_). NMR ^13^C{^1^H} (100.9 MHz, DMSO-d_6_, 25 °C) δC, ppm.: 137.1, 123.3, 122.7, 69.7, 68.3, 50.3, 49.3, 46.3, 30.1. IR (KBr) ν_max_, cm^−1^: ν 3086 m (С_Ar_-H), ν_as_ 2949 m. (СH_2_), ν_as_ 2102 s (-N_3_), ν 1627 s (С=N), ν 1551 m (C=C), ν 1458 s (С-N), ν_as_ 1086 m (C-O-C). HRMS-ESI: found to be *m*/*z* 202.1194 [M-2Br]^2+^; calculated C_17_H_28_N_10_O_2_^2+^ 202.1193.

General procedure for preparation of triazole containing bis-imidazolium derivatives **14** and **15**.

To 0.5 mmol of compound **12′a** or **12′b** or **12′c** or **13′a** or **13′b** or **13′c**, we added 0.63 mmol (for compound **12′d** or **13′d**—1.26 mmol) of triethylene glycol methyl propargyl ether, 0.03 mmol (for compound **12′d** or **13′d**—0.06 mmol) of CuI(I) and 6.25 mmol (for compound **12′d** or **13′d**—12.5 mmol) of triethylamine. The resulting mixture was dissolved in 3 mL of acetonitrile and stirred at 60 °C for 6 h. Then, the solvent was evaporated and the resulting residue was dissolved in water (10 mL). To remove copper, the aqueous solution was passed through a layer of amberlite IRA-67. The filtrate was then washed with diethyl ether (2 × 15 mL). After adding water, the target product was obtained as a yellow viscous oil.

1-(3-(4-(2,5,8,11-tetraoxadodecyl)-1H-1,2,3-triazol-1-yl)propyl)-3-(3-(1-butyl-1H-imidazol-3-ium-3-yl)propyl)-1H-imidazol-3-ium dibromide (**14a**). Yield 0.27 g (80%). NMR ^1^H (400 MHz, DMSO-d_6_, 25 °C) δH, ppm.: 9.42 (s, 1H, ImH), 9.38 (s, 1H, ImH), 8.19 (s, 1H, TrzH), 7.89 (s, 1H, ImH), 7.87 (s, 1H, ImH), 7.86 (s, 1H, ImH), 7.84 (s, 1H, ImH), 4.51 (s, 2H, CH_2_C_trz_), 4.47 (t, *J* = 6.6 Hz, 2H, CH_2_N_trz_), 4.27 (m, 6H, CH_2_Im), 4.19 (t, *J* = 7.2 Hz, 2H, CH_2_Im), 3.58–3.47 (m, 10H, CH_2_O), 3.43–3.39 (m, 2H, CH_2_O), 3.21 (s, 3H, OCH_3_), 2.49–2.39 (m, 4H, CH_2_), 1.84–1.72 (m, 2H, CH_2_), 1.31–1.22 (m, 2H, CH_2_), 0.90 (t, *J* = 7.3 Hz, 2H, CH_3_). NMR ^13^C{^1^H} (100.9 MHz, DMSO-d_6_, 25 °C) δC, ppm.: 144.0, 136.6, 136.3, 124.1, 122.6, 122.5, 122.5, 122.4, 71.3, 69.8, 69.8, 69.7, 69.6, 69.0, 63.5, 58.1, 48.7, 46.6, 46.5, 45.9, 45.8, 31.3, 29.6, 29.4, 18.8, 13.3. IR (KBr) ν_max_, cm^−1^: ν 3083 m (С_Ar_-H), ν_as_ 2934 m (СH_2_), ν 1644 m (С=N), ν 1564 s (C=C), ν 1461 m (С-N), ν_as_ 1099 s (C-O-C), ν_s_ 848 m (C-O-C). HRMS-ESI: found to be *m*/*z* 259.6764 [M-2Br]^2+^; calculated for: C_26_H_45_N_7_O_4_^2+^ 259.6761.

1-(3-(4-(2,5,8,11-tetraoxadodecyl)-1H-1,2,3-triazol-1-yl)propyl)-3-(3-(1-octyl-1H-imidazol-3-ium-3-yl)propyl)-1H-imidazol-3-ium dibromide (**14b**). Yield 0.31 g (82%). NMR ^1^H (400 MHz, DMSO-d_6_, 25 °C) δH, ppm.: 9.39 (br d, 2H, ImH), 8.18 (s, 1H, TrzH), 7.92–7.79 (m, 4H, ImH), 4.51 (s, 2H, CH_2_C_trz_), 4.47 (t, *J* = 6.4 Hz, 2H, CH_2_N_trz_), 4.33–4.22 (m, 6H, CH_2_Im), 4.18 (t, *J* = 7.0 Hz, 2H, CH_2_Im), 3.58–3.46 (m, 10H, CH_2_O), 3.42–3.41 (m, 2H, CH_2_O), 3.21 (s, 3H, OCH_3_), 2.48–2.39 (m, 4H, CH_2_), 1.85–1.74 (m, 2H, CH_2_), 1.30–1.18 (m, 10H, CH_2_), 0.84 (t, *J* = 6.3 Hz, 3H, CH_3_). NMR ^13^C{^1^H} (100.9 MHz, DMSO-d_6_, 25 °C) δC, ppm.: 144.5, 137.1, 136.7, 124.6, 123.0, 123.0, 123.0, 122.9, 71.7, 70.2, 70.2, 70.0, 69.5, 64.0, 58.5, 49.4, 47.0, 46.9, 46.3, 46.3, 31.6, 30.1, 29.9, 29.8, 29.0, 28.8, 26.0, 22.5, 14.4. IR (KBr) ν_max_, cm^−1^: ν 3071 m (С_Ar_-H), ν_as_ 2929 m (СH_2_), ν 1671 m (С=N), ν 1561 s (C=C), ν 1458 m (С-N), ν_as_ 1101 s (C-O-C), ν_s_ 849 m (C-O-C). HRMS-ESI: found to be *m*/*z* 287.7040 [M-2Br]^2+^; calculated for: C_30_H_53_N_7_O_4_^2+^ 287.7074.

1-(3-(4-(2,5,8,11-tetraoxadodecyl)-1H-1,2,3-triazol-1-yl)propyl)-3-(3-(1-tetradecyl-1H-imidazol-3-ium-3-yl)propyl)-1H-imidazol-3-ium dibromide (**14c**). Yield 0.34 g (83%). NMR ^1^H (400 MHz, DMSO-d_6_, 25 °C) δH, ppm.: 9.30 (s, 1H, ImH), 9.28 (s, 1H, ImH), 8.15 (s, 1H, TrzH), 7.93–7.78 (m, 4H, ImH), 4.51 (s, 2H, CH_2_C_trz_), 4.46 (t, *J* = 6.6 Hz, 2H, CH_2_N_trz_), 4.30–4.20 (m, 6H, CH_2_Im), 4.17 (t, *J* = 7.2 Hz, 2H, CH_2_Im), 3.59–3.46 (m, 10H, CH_2_O), 3.44–3.39 (m, 2H, CH_2_O), 3.22 (s, 3H, OCH_3_), 2.48–2.38 (m, 4H, CH_2_), 1.85–1.74 (m, 2H, CH_2_), 1.31–1.17 (m, 22H, CH_2_), 0.85 (t, *J* = 6.4 Hz, 3H, CH_3_). NMR ^13^C{^1^H} (100.9 MHz, DMSO-d_6_, 25 °C) δC, ppm.: 144.1, 136.6, 136.2, 124.1, 122.6, 122.5, 122.5, 122.4, 71.3, 69.8, 69.8, 69.7, 69.6, 69.1, 63.5, 58.1, 49.0, 46.6, 46.5, 45.9, 31.3, 29.6, 29.4, 29.4, 29.1, 29.1, 29.0, 29.0, 28.9, 28.7, 28.4, 25.6, 22.1, 14.0. IR (KBr) ν_max_, cm^−1^: ν 3094 m (С_Ar_-H), ν_as_ 2924 m (СH_2_), ν 1644 m (С=N), ν 1565 s (C=C), ν 1458 m (С-N), ν_as_ 1098 s (C-O-C), ν_s_ 848 m (C-O-C). HRMS-ESI: found to be *m*/*z*: 329.7544 [M-2Br]^2+^; calculated for: C_36_H_65_N_7_O_4_^2+^ *m*/*z*: 329.7544.

3,3′-(propane-1,3-diyl)bis(1-(3-(4-(2,5,8,11-tetraoxadodecyl)-1H-1,2,3-triazol-1-yl)propyl)-1H-imidazol-3-ium) dibromide (**14d**). Yield 0.36 g (79%). NMR ^1^H (400 MHz, DMSO-d_6_, 25 °C) δH, ppm.: 9.33 (s, 2H, ImH), 8.17 (s, 2H, CH_2_N_trz_), 7.84 (s, 4H, ImH), 4.51 (s, 4H, CH_2_C_trz_), 4.47 (t, *J* = 6.6 Hz, 4H, CH_2_N_trz_), 4.32–4.21 (m, 8H, CH_2_Im), 3.57–3.46 (m, 20H, CH_2_O), 3.43–3.39 (m, 4H, CH_2_O), 3.22 (s, 6H, OCH_3_), 2.47–2.39 (m, 6H, CH_2_). NMR ^13^C{^1^H} (100.9 MHz, DMSO-d_6_, 25 °C) δC, ppm.: 144.1, 136.6, 124.1, 122.5, 71.3, 69.8, 69.7, 69.6, 69.0, 63.5, 58.0, 46.6, 46.4, 45.8, 29.6, 29.3. IR (KBr) ν_max_, cm^−1^: ν 3070 m (С_Ar_-H), ν_as_ 2878 m (СH_2_), ν 1676 m (С=N), ν 1564 s (C=C), ν 1457 m (С-N), ν_as_ 1098 s (C-O-C), ν_s_ 849 m (C-O-C). HRMS-ESI: found to be *m*/*z* 374.2294 [M-2Br]^2+^; calculated for: C_35_H_60_N_10_O_8_^2+^ 374.2292.

1-(2-(2-(4-(2,5,8,11-tetraoxadodecyl)-1H-1,2,3-triazol-1-yl)ethoxy)ethyl)-3-(3-(1-butyl-1H-imidazol-3-ium-3-yl)propyl)-1H-imidazol-3-ium dibromide (**15a**). Yield 0.28 g (79%). NMR ^1^H (400 MHz, DMSO-d_6_, 25 °C) δH, ppm.: 9.40 (s, 1H, ImH), 9.22 (s, 1H, ImH), 8.08 (s, 1H, TrzH), 7.87 (s, 1H, ImH), 7.86 (s, 1H, ImH), 7.80 (s, 1H, ImH), 7.66 (s, 1H, ImH), 4.53 (t, *J* = 4.9 Hz, 2H, CH_2_C_trz_), 4.49 (s, 2H, CH_2_N_trz_), 4.34 (t, *J* = 4.6 Hz, 2H, CH_2_Im), 4.31–4.24 (m, 4H, CH_2_Im), 4.18 (t, *J* = 7.2 Hz, 2H, CH_2_Im), 3.84 (t, *J* = 4.9 Hz, 2H, CH_2_O), 3.77 (t, *J* = 4.7 Hz, 2H, CH_2_O), 3.58–3.45 (m, 10H, CH_2_O), 3.43–3.38 (m, 2H, CH_2_O), 3.21 (s, 3H, OCH_3_), 2.48–2.39 (m, 2H, CH_2_), 1.83–1.73 (m, 2H, CH_2_), 1.32–1.23 (m, 2H, CH_2_), 0.89 (t, *J* = 7.3 Hz, 3H, CH_3_). NMR ^13^C{^1^H} (100.9 MHz, DMSO-d_6_, 25 °C) δC, ppm.: 143.8, 136.5, 136.3, 124.3, 122.8, 122.6, 122.4, 122.1, 71.3, 69.8, 69.8, 69.7, 69.6, 69.0, 68.5, 67.7, 63.5, 58.1, 49.1, 48.7, 48.7, 45.9, 31.3, 29.5, 18.9, 13.4. IR (KBr) ν_max_, cm^−1^: ν 3082 m (С_Ar_-H), ν_as_ 2933 m (СH_2_), ν 1642 m (С=N), ν 1564 s (C=C), ν 1460 m (С-N), ν_as_ 1101 s (C-O-C), ν_s_ 848 m (C-O-C). HRMS-ESI: found to be m/z 274.6812 [M-2Br]^2+^; calculated for: C_27_H_47_N_7_O_5_^2+^ 274.6814.

1-(2-(2-(4-(2,5,8,11-tetraoxadodecyl)-1H-1,2,3-triazol-1-yl)ethoxy)ethyl)-3-(3-(1-octyl-1H-imidazol-3-ium-3-yl)propyl)-1H-imidazol-3-ium dibromide (**15b**). Yield 0.29 g (77%). NMR ^1^H (400 MHz, DMSO-d_6_, 25 °C) δH, ppm.: 9.37 (s, 1H, ImH), 9.19 (s, 1H, ImH), 8.08 (s, 1H, TrzH), 7.87 (br s, 2H, ImH), 7.80 (s, 1H, ImH), 7.67 (s, 1H, ImH), 4.54 (t, *J* = 4.8 Hz, 2H, CH_2_N_trz_), 4.51 (s, 2H, CH_2_C_trz_), 4.36 (t, *J* = 4.3 Hz, 2H, CH_2_Im), 4.32–4.25 (m, 4H, CH_2_Im), 4.19 (t, *J* = 7.2 Hz, 2H, CH_2_Im), 3.86 (t, *J* = 4.9 Hz, 2H, CH_2_O), 3.79 (t, *J* = 4.5 Hz, 2H, CH_2_O), 3.59–3.48 (m, 10H, CH_2_O), 3.44–3.41 (m, 2H, CH_2_O), 3.23 (s, 3H, OCH_3_), 2.49–2.39 (m, 2H, CH_2_), 1.86–1.75 (m, 2H, CH_2_), 1.33–1.17 (m, 10H, CH_2_), 0.86 (t, *J* = 6.6 Hz, 3H, CH_3_). NMR ^13^C{^1^H} (100.9 MHz, DMSO-d_6_, 25 °C) δC, ppm.: 143.7, 136.5, 136.3, 124.3, 122.8, 122.5, 122.4, 122.1, 71.2, 69.8, 69.7, 69.7, 69.5, 69.0, 68.5, 67.7, 63.5, 58.0, 49.1, 48.9, 48.7, 45.8, 31.2, 29.5, 29.3, 28.5, 28.4, 25.5, 22.0, 13.9. IR (KBr) ν_max_, cm^−1^: ν 3079 m (С_Ar_-H), ν_as_ 2865 m (СH_2_), ν 1642 m (С=N), ν 1576 s (C=C), ν 1465 m (С-N), ν_as_ 1093 s (C-O-C), ν_s_ 851 m (C-O-C). HRMS-ESI: found to be *m*/*z* 302.7113 [M-2Br]^2+^; calculated for: C_31_H_55_N_7_O_5_^2+^ 302.7127.

1-(2-(2-(4-(2,5,8,11-tetraoxadodecyl)-1H-1,2,3-triazol-1-yl)ethoxy)ethyl)-3-(3-(1-tetradecyl-1H-imidazol-3-ium-3-yl)propyl)-1H-imidazol-3-ium dibromide (**15c**). Yield 0.31 g (72%). NMR ^1^H (400 MHz, DMSO-d_6_, 25 °C) δH, ppm.: 9.35 (s, 1H, ImH), 9.17 (s, 1H, ImH), 8.07 (s, 1H, TrzH), 7.85 (br s, 2H, ImH), 7.79 (s, 1H, ImH), 7.65 (s, 1H, ImH), 4.53 (t, *J* = 5.2 Hz, 2H, CH_2_N_trz_), 4.50 (s, 2H, CH_2_C_trz_), 4.34 (t, *J* = 4.2 Hz, 2H, CH_2_Im), 4.31–4.23 (m, 4H, CH_2_Im), 4.17 (t, *J* = 7.2 Hz, 2H, CH_2_Im), 3.84 (t, *J* = 4.8 Hz, 2H, CH_2_O), 3.77 (t, *J* = 4.5 Hz, 2H, CH_2_O), 3.59–3.46 (m, 10H, CH_2_O), 3.44–3.39 (m, 2H), 3.21 (s, 3H, OCH3), 2.46–2.38 (m, 2H, CH_2_), 1.88–1.75 (m, 2H, CH2), 1.40–1.13 (m, 22H, CH_2_), 0.85 (t, *J* = 6.6 Hz, 3H, CH_3_). NMR ^13^C{^1^H} (100.9 MHz, DMSO-d_6_, 25 °C) δC, ppm.: 143.8, 136.5, 136.3, 124.3, 122.8, 122.6, 122.4, 122.1, 71.3, 69.8, 69.7, 69.6, 69.1, 68.5, 67.7, 63.5, 58.1, 49.1, 49.0, 48.8, 45.9, 31.3, 29.5, 29.4, 29.1, 29.1, 29.0, 28.9, 28.8, 28.5, 25.6, 22.1, 14.0. IR (KBr) ν_max_, cm^−1^: ν 3107 m (С_Ar_-H), ν_as_ 2906 m (СH_2_), ν 1651 m (С=N), ν 1565 s (C=C), ν 1456 m (С-N), ν_as_ 1099 s (C-O-C), ν_s_ 847 m (C-O-C). HRMS-ESI: found to be m/z 344.7596 [M-2Br]^2+^; calculated for: C_37_H_67_N_7_O_5_^2+^ 344.7596.

3,3′-(propane-1,3-diyl)bis(1-(2-(2-(4-(2,5,8,11-tetraoxadodecyl)-1H-1,2,3-triazol-1-yl)ethoxy)ethyl)-1H-imidazol-3-ium) dibromide (**15d**). Yield 0.41 g (85%). NMR ^1^H (400 MHz, DMSO-d_6_, 25 °C) δH, ppm.: 9.16 (s, 2H, ImH), 8.06 (s, 2H, TrzH), 7.79 (s, 2H, ImH), 7.65 (s, 2H, ImH), 4.53 (t, *J* = 4.9 Hz, 4H, CH_2_N_trz_), 4.50 (s, 4H, CH_2_C_trz_), 4.34 (t, *J* = 4.5 Hz, 4H, CH_2_Im), 4.28 (t, *J* = 7.0 Hz, 4H, CH_2_Im), 3.84 (t, *J* = 5.0 Hz, 4H, CH_2_O), 3.77 (t, *J* = 4.6 Hz, 4H, CH_2_O), 3.59–3.48 (m, 20H, CH_2_O), 3.44–3.39 (m, 4H, CH_2_O), 3.21 (s, 6H, OCH_3_), 2.47–2.37 (m, 2H, CH_2_). NMR ^13^C{^1^H} (100.9 MHz, DMSO-d_6_, 25 °C) δC, ppm.: 143.8, 136.5, 124.2, 122.8, 122.1, 71.2, 69.8, 69.7, 69.5, 69.0, 68.5, 67.7, 63.5, 58.0, 49.1, 48.8, 45.9, 29.4. IR (KBr) ν_max_, cm^−1^: ν 3082 m (С_Ar_-H), ν_as_ 2933 m (СH_2_), ν 1642 m (С=N), ν 1564 s (C=C), ν 1460 m (С-N), ν_as_ 1101 s (C-O-C), ν_s_ 848 m (C-O-C). HRMS-ESI: found to be *m*/*z* 404.2396 [M-2Br]^2+^; calculated for: C_37_H_64_N_10_O_10_^2+^ 404.2398.

### 3.4. Fluorescence Spectroscopy

Fluorescence emission spectra were recorded on a Jobin Yvon Horiba Fluorolog-3 (HORIBA Jobin Yvon SAS, Longjumeau, France). A 1 cm quartz cuvette was used. For the fluorescence measurements using pyrene as a polarity probe, the concentration of pyrene used was 1 μM. The emission spectra of pyrene were recorded in the wavelength range of 350–430 nm at an excitation wavelength of 334 nm using excitation and emission slit widths of 2.5 nm. The fluorescence of BSA was measured at an excitation wavelength of 295 nm with a 3 nm slit; the emission spectra were recorded in the range of 305 to 450 nm.

### 3.5. Dynamic and Electrophoretic Light Scattering

Dynamic and electrophoretic light scattering measurements were performed at 298.15 K on light scattering apparatus (Zetasizer Nano, Malvern Instruments Ltd., Malvern, Worcestershire, UK). Appropriate amounts of the surfactants (700 µM) were added in aliquots to the BSA solution (10 µM) in the cuvette.

## 4. Conclusions

In this work, dicationic Gemini surfactants with different symmetric and asymmetric substituents were synthesized for the first time using the click-reaction method. The physicochemical properties of the synthesized surfactants were analyzed. The effect of Gemini surfactant structure on the CAC value was analyzed. It was shown that the largest surfactant/BSA binding constant was obtained for the asymmetric hydrophobic **14c**. Using the temperature dependences of the binding constants according to the van-Hoff method, the interaction mechanisms of the synthesized Gemini surfactants with BSA were determined. The data on the surfactants’ interaction with BSA indicate that the choice of substituent allows changing of the mechanism of surfactant/BSA binding from hydrophobic to electrostatic for **15c**. Additionally, the change in the interaction mechanism in the **15c**/BSA system was confirmed by the DLS data. According to these data, in the presence of **15c**, the hydrodynamic diameter of the formed aggregates with BSA is 10 times larger than for all other surfactants. Thus, the physicochemical properties of alkyl surfactants can be fine-tuned depending on the choice of alkyl radical to solve a particular physicochemical problem.

## Figures and Tables

**Table 1:** Critical aggregation concentration (CAC) and dynamic and electrophoretic light scattering values of surfactants.

Surfactant	CAC ^1^, mM	Concentration, mM	d, nm	PDI	ζ, mV
**15b**	3.2	3.5	495 ± 25	0.47	+29 ± 4
**15c**	1.1	1.2	445 ± 10	0.34	+35 ± 1
**14b**	2.7	3.0	410 ± 32	0.53	+16 ± 2
**14c**	0.9	1.1	210 ± 20	0.45	+36 ± 3

^1^ [pyrene] 1 μM.

**Table 2:** The binding and thermodynamic parameters of binding between surfactants with BSA.

BSA *	Temperature, K	$K_{\mathrm{sv}},{\;M}^{-1}$	$K_{b},{\;M}^{-1}$	ΔH, kJ mol^−1^	ΔS, J mol^−1^ K^−1^	n
**14a**	298.15	0.37$\;\times \;10^{3}$	0.73$\;\times \;10^{3}$	24	136	1.1
	303.15	0.42$\;\times \;10^{3}$	0.90$\;\times \;10^{3}$			
	310.15	0.48$\;\times \;10^{3}$	1.07$\;\times \;10^{4}$			
**14b**	298.15	1.70$\;\times \;10^{4}$	2.0$7\;\times \;10^{4}$	27	174	1.1
	303.15	1.62$\;\times \;10^{4}$	2.55$\;\times \;10^{4}$			
	310.15	1.59$\;\times \;10^{4}$	3.18$\;\times \;10^{4}$			
**14c**	298.15	4.88$\;\times \;10^{4}$	4.49$\;\times \;10^{4}$	39	219	1.3
		1.36$\;\times \;10^{4}$	1.48$\;\times \;10^{4}\;$ ^b^			0.9
		4.54$\;\times \;10^{4}$	6.39$\;\times \;10^{4\;}$ ^a^			1.2
	303.15	4.68$\;\times \;10^{4}$	6.05$\;\times \;10^{4}$			
	310.15	4.45$\;\times \;10^{4}$	8.25$\;\times \;10^{4}$			
**14d**	298.15	1.13$\;\times \;10^{3}$	3.48$\;\times \;10^{4}$	19	152	1.2
	303.15	1.15$\;\times \;10^{3}$	4.24$\;\times \;10^{4}$			
	310.15	1.13$\;\times \;10^{3}$	4.74$\;\times \;10^{4}$			
**15a**	298.15	0.29$\;\times \;10^{3}$	0.79$\;\times \;10^{3}$	8	82	1.1
	303.15	0.32$\;\times \;10^{3}$	0.84$\;\times \;10^{3}$			
	310.15	0.35$\;\times \;10^{3}$	0.89$\;\times \;10^{3}$			
**15b**	298.15	1.34$\;\times \;10^{3}$	1.0$4\;\times \;10^{4}$	58	270	1.4
	303.15	1.29$\;\times \;10^{3}$	1.43$\;\times \;10^{4}$			
	310.15	1.22$\;\times \;10^{3}$	2.54$\;\times \;10^{4}$			
**15c**	298.15	4.82$\;\times \;10^{3}$	1.0$9\;\times \;10^{4}$	−5	61	1.1
	303.15	4.12$\;\times \;10^{3}$	1.$04\;\times \;10^{4}$			
	310.15	3.54$\;\times \;10^{3}$	1.$01\;\times \;10^{4}$			
**15d**	298.15	1.4$0\;\times \;10^{3}$	1.0$\;\times \;10^{4}$	25	162	1.3
	303.15	1.27$\;\times \;10^{3}$	1.3$\;\times \;10^{4}$			
	310.15	1.03$\;\times \;10^{3}$	1.5$\;\times \;10^{4}$			

* [BSA] 10 μM, [surfactant] 0–700 μM. ^a^ **14c** at pH 6 (phosphate buffer, 20 mM). ^b^ **14c** at pH 9 (Tris buffer, 20 mM).

**Table 3:** Dynamic and electrophoretic light scattering data for surfactant/BSA systems.

BSA *	d, nm	PDI	Zeta-Potential, mV
**-**	10 ± 1	0.25	−6.7 ± 0.4
**14a**	9 ± 1	0.24	−10 ± 0.8
**14b**	11 ± 1	0.36	−7.7 ± 0.2
**14c**	16 ± 1	0.36	+20.3 ± 0.9
**14d**	9 ± 1	0.29	−9.3 ± 0.2
**15a**	8 ± 1	0.21	−14.2 ± 0.7
**15b**	11 ± 1	0.36	−11.6 ± 0.3
**15c**	151 ± 2	0.34	+8.3 ± 1.3
**15d**	10 ± 1	0.34	−9.7 ± 0.3

* [BSA] 10 μM, [surfactant] 700 μM 25 mM Tris-HCl buffer solution with pH 7.4.

## Data Availability

The data are contained within the article or Supplementary Materials.

## Supplementary Materials

The following supporting information can be downloaded at: https://www.mdpi.com/article/10.3390/molecules29225420/s1, Figure S1: Fragments of ^1^H NMR spectra of compound **12** (A) and compound **12′a** (B) (400 MHz, DMSO-d_6_, 25 °C); Figure S2: Fragments of ^1^H NMR spectra of compound **12′a** (A) and compound **14** (B) (400 MHz, DMSO-d_6_, 25 °C); Figure S3: NMR ^1^H (a), ^13^C{^1^H}{^1^H} (b), FTIR (c) and ESI (d) spectra *of 3-(3-azidopropyl)-1-(3-bromopropyl)-^1^H-imidazolium bromide* (**12**); Figure S4. NMR ^1^H (a), ^13^C{^1^H}{^1^H} (b), FTIR (c) and ESI (d) spectra of *3-(2-(2-azidoethoxy)ethyl)-1-(3-bromopropyl)-^1^H-imidazolium bromide* (**13**); Figure S5. NMR ^1^H (a), ^13^C{^1^H}{^1^H} (b), FTIR (c) and ESI (d) spectra of *3-(3-azidopropyl)-1-(3-(1-butyl-^1^H-imidazol-3-yl)propyl)-^1^H-imidazolium dibromide* (**12a**); Figure S6. NMR ^1^H (a), ^13^C{^1^H} (b), FTIR (c) and ESI (d) spectra of *3-(3-azidopropyl)-1-(3-(1-octyl-^1^H-imidazol-3-yl)propyl)-^1^H-imidazolium dibromide* (**12b**); Figure S7. NMR ^1^H (a), ^13^C{^1^H} (b), FTIR (c) and ESI (d) spectra of *3-(3-azidopropyl)-1-(3-(1-tetradecyl-^1^H-imidazol-3-yl)propyl)-^1^H-imidazolium dibromide* (**12c**); Figure S8. NMR ^1^H (a), ^13^C{^1^H} (b), FTIR (c) and ESI (d) spectra of *3-(3-azidopropyl)-1-(3-(1-tetradecyl-^1^H-imidazol-3-yl)propyl)-^1^H-imidazolium dibromide* (**12d**); Figure S9. NMR ^1^H (a), ^13^C{^1^H} (b), FTIR (c) and ESI (d) spectra of *1-(2-(2-azidoethoxy)ethyl)-3-(3-(1-butyl-^1^H-imidazol-3-yl)propyl)-^1^H-imidazolium dibromide* (**13a**); Figure S10. NMR ^1^H (a), ^13^C{^1^H} (b), FTIR (c) and ESI (d) spectra of *1-(2-(2-azidoethoxy)ethyl)-3-(3-(1-octyl-^1^H-imidazol-3-yl)propyl)-^1^H-imidazolium dibromide* (**13b**); Figure S11. NMR ^1^H (a), ^13^C{^1^H} (b), FTIR (c) and ESI (d) spectra of *1-(2-(2-azidoethoxy)ethyl)-3-(3-(1-tetradecyl-^1^H-imidazol-3-yl)propyl)-^1^H-imidazolium dibromide* (**13c**); Figure S12. NMR ^1^H (a), ^13^C{^1^H} (b), FTIR (c) and ESI (d) spectra of *3,3′-(propane-1,3-diyl)bis(1-(2-(2-azidoethoxy)ethyl)-^1^H-imidazol-3-ium) dibromide* (**13d**); Figure S13. NMR ^1^H (a), ^13^C{^1^H} (b), FTIR (c) and ESI (d) spectra of *1-(3-(4-(2,5,8,11-tetraoxadodecyl)-^1^H-1,2,3-triazol-1-yl)propyl)-3-(3-(1-butyl-^1^H-imidazol-3-ium-3-yl)propyl)-^1^H-imidazol-3-ium dibromide* (**14a**); Figure S14. NMR ^1^H (a), ^13^C{^1^H} (b), FTIR (c) and ESI (d) spectra of *1-(3-(4-(2,5,8,11-tetraoxadodecyl)-^1^H-1,2,3-triazol-1-yl)propyl)-3-(3-(1-octyl-^1^H-imidazol-3-ium-3-yl)propyl)-^1^H-imidazol-3-ium dibromide* (**14b**); Figure S15. NMR ^1^H (a), ^13^C{^1^H} (b), FTIR (c) and ESI (d) spectra of *1-(3-(4-(2,5,8,11-tetraoxadodecyl)-^1^H-1,2,3-triazol-1-yl)propyl)-3-(3-(1-tetradecyl-^1^H-imidazol-3-ium-3-yl)propyl)-^1^H-imidazol-3-ium dibromide* (**14c**); Figure S16. NMR ^1^H (a), ^13^C{^1^H} (b), FTIR (c) and ESI (d) spectra of 3*,3′-(propane-1,3-diyl)bis(1-(3-(4-(2,5,8,11-tetraoxadodecyl)-^1^H-1,2,3-triazol-1-yl)propyl)-^1^H-imidazol-3-ium) dibromide* (**14d**); Figure S17. NMR ^1^H (a), ^13^C{^1^H} (b), FTIR (c) and ESI (d) spectra of *1-(2-(2-(4-(2,5,8,11-tetraoxadodecyl)-^1^H-1,2,3-triazol-1-yl)ethoxy)ethyl)-3-(3-(1-butyl-^1^H-imidazol-3-ium-3-yl)propyl)-^1^H-imidazol-3-ium dibromide* (**15a**); Figure S18. NMR ^1^H (a), ^13^C{^1^H} (b), FTIR (c) and ESI (d) spectra of *1-(2-(2-(4-(2,5,8,11-tetraoxadodecyl)-^1^H-1,2,3-triazol-1-yl)ethoxy)ethyl)-3-(3-(1-octyl-^1^H-imidazol-3-ium-3-yl)propyl)-^1^H-imidazol-3-ium dibromide* (**15b**); Figure S19. NMR ^1^H (a), ^13^C{^1^H} (b), FTIR (c) and ESI (d) spectra of *1-(2-(2-(4-(2,5,8,11-tetraoxadodecyl)-^1^H-1,2,3-triazol-1-yl)ethoxy)ethyl)-3-(3-(1-tetradecyl-^1^H-imidazol-3-ium-3-yl)propyl)-^1^H-imidazol-3-ium dibromide* (**15c**); Figure S20. NMR ^1^H (a), ^13^C{^1^H} (b), FTIR (c) and ESI (d) spectra of *3,3′-(propane-1,3-diyl)bis(1-(2-(2-(4-(2,5,8,11-tetraoxadodecyl)-^1^H-1,2,3-triazol-1-yl)ethoxy)ethyl)-^1^H-imidazol-3-ium) dibromide* (**15d**); Figure S21. The ratio of the fluorescence intensities of the first (372 nm) and third (385 nm) pyrene emission peaks as a function of the concentration of amphiphilic molecules for binary surfactant/pyrene, [pyrene] = 1 μM; Figure S22. Emission spectra of BSA system in the absence and presence of various concentrations of **14d** (A), **14a** (B), **14b** (C) and **14c** (D) in 25 mM Tris-HCl buffer solution with pH 7.4, [BSA] 10 μM; Figure S23. Emission spectra of BSA system in the absence and presence of various concentrations of **15d** (A), **15a** (B), **15b** (C) and **15c** (D) in 25 mM Tris-HCl buffer solution with pH 7.4, [BSA] 10 μM. Figure S24. Dynamic light scattering particle size distribution of BSA (10 μM) suspended in 25 mM Tris-HCl buffer solution with pH 7.4; Figure S25. Dynamic light scattering particle size distribution of BSA (10 μM) in presence of surfactant (700 μM) ((A) **14a**; (B) **14b**; (C) **14c**; (D) **14d**) in 25 mM Tris-HCl buffer solution with pH 7.4; Figure S26. Dynamic light scattering particle size distribution of BSA (10 μM) in presence of surfactant (700 μM) ((A) **15a**; (B) **15b**; (C) **15c**; (D) **15d**) in 25 mM Tris-HCl buffer solution with pH 7.4.
