# Supplementary material for: New Asymmetric Gemini Triazole Surfactants with a Polar Triethylene Glycol Fragment: Synthesis and Physico-Chemical Properties
Source: Molecules. 2024 Nov 17;29(22):5420. doi: 10.3390/molecules29225420 (PMC11597725; doi:10.3390/molecules29225420)
Supplement: Supplementary file 1 [file molecules-29-05420-s001.zip › molecules-3289640-supplementary.pdf]

New asymmetric gemini triazole surfactants with a polar triethylene glycol fragment: synthesis and physical-chemical properties

Ilshat Bogdanov [1], Diana Mironova [1]*, Elza Sultanova [1], Vladimir Burilov [1], Svetlana Solovieva [2], Igor Antipin [1]

[1] Department of Organic and Medicinal Chemistry, Kazan Federal University, Kremlyovskaya str. 18, 420008, Kazan, Russian Federation
[2] Arbuzov Institute of Organic and Physical Chemistry, FRC Kazan Scientific Center of RAS, Arbuzov str. 8, 420088, Kazan, Russian Federation
* Correspondence: mir_din@mail.ru; Tel.: +7(843)2337344

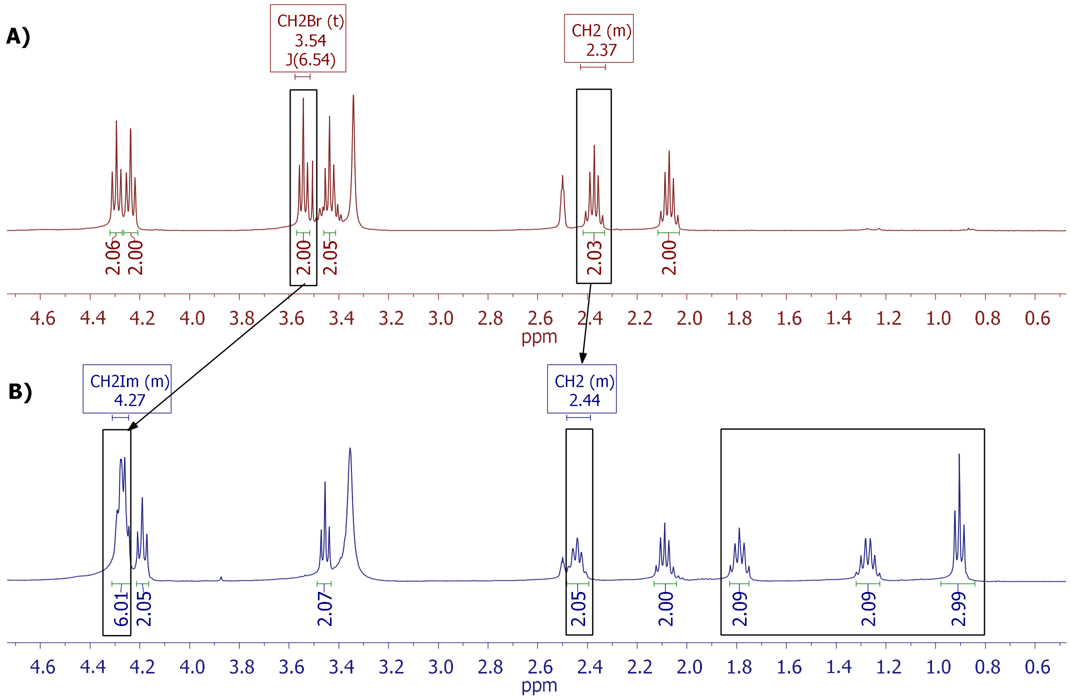

Figure S1. Fragments of ^{1}H NMR spectra of compound **12** (A) and compound **12'a** (B) (400 MHz, DMSO-d$_6$, 25°C).

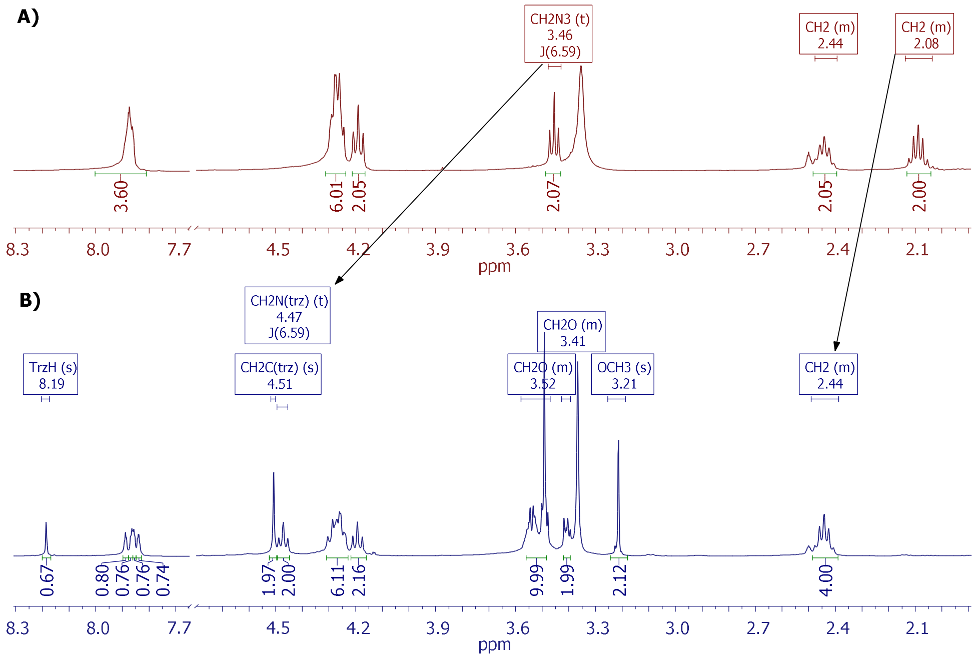

Figure S2. Fragments of ^{1}H NMR spectra of compound **12'a** (A) and compound **14** (B) (400 MHz, DMSO-d$_6$, 25°C).

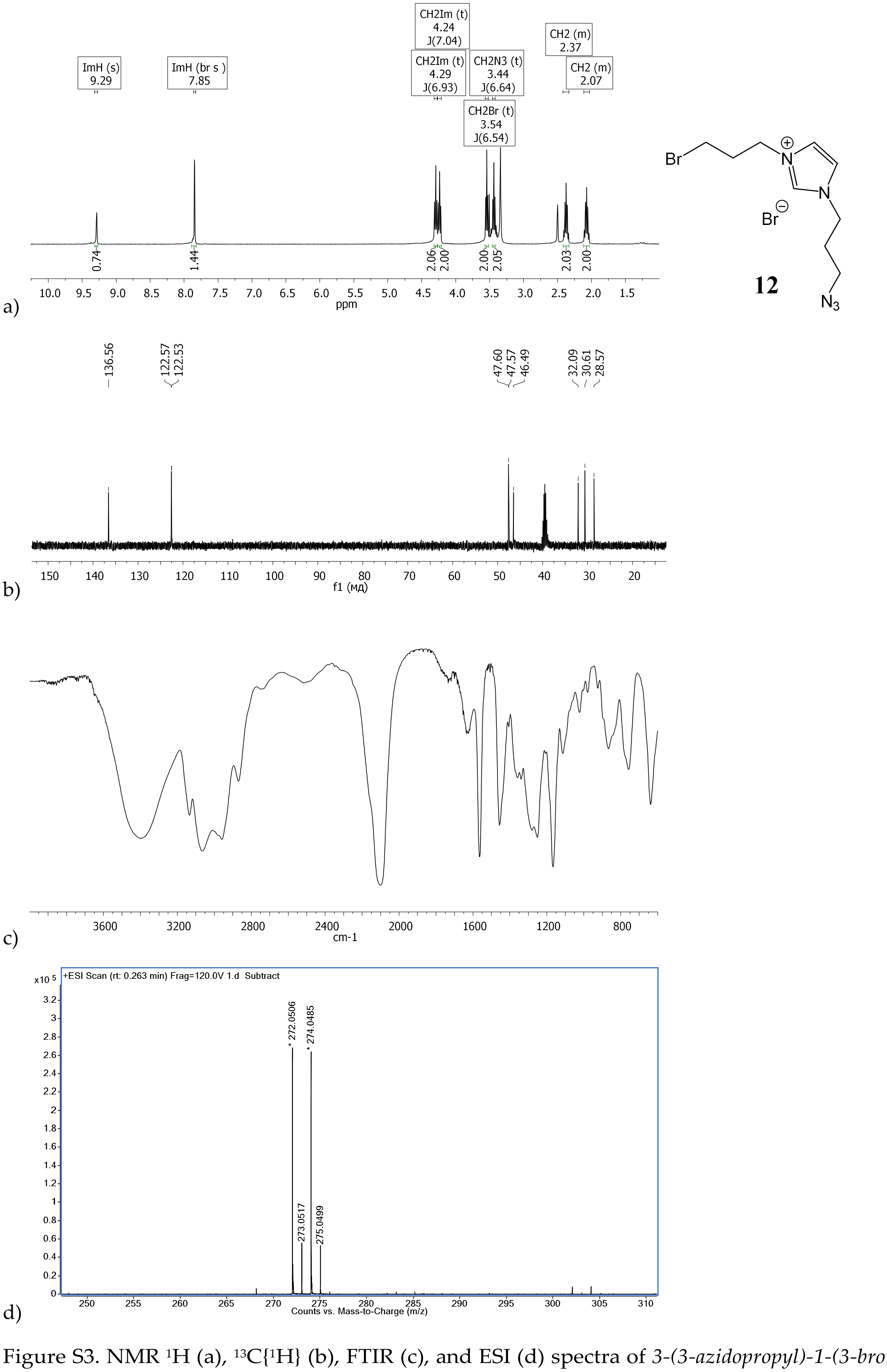

Figure S3. NMR ^{1}H (a), $^{13}C\{^{1}H\}$ (b), FTIR (c), and ESI (d) spectra of *3-(3-azidopropyl)-1-(3-bromopropyl)-1H-imidazolium bromide* ***(12)***

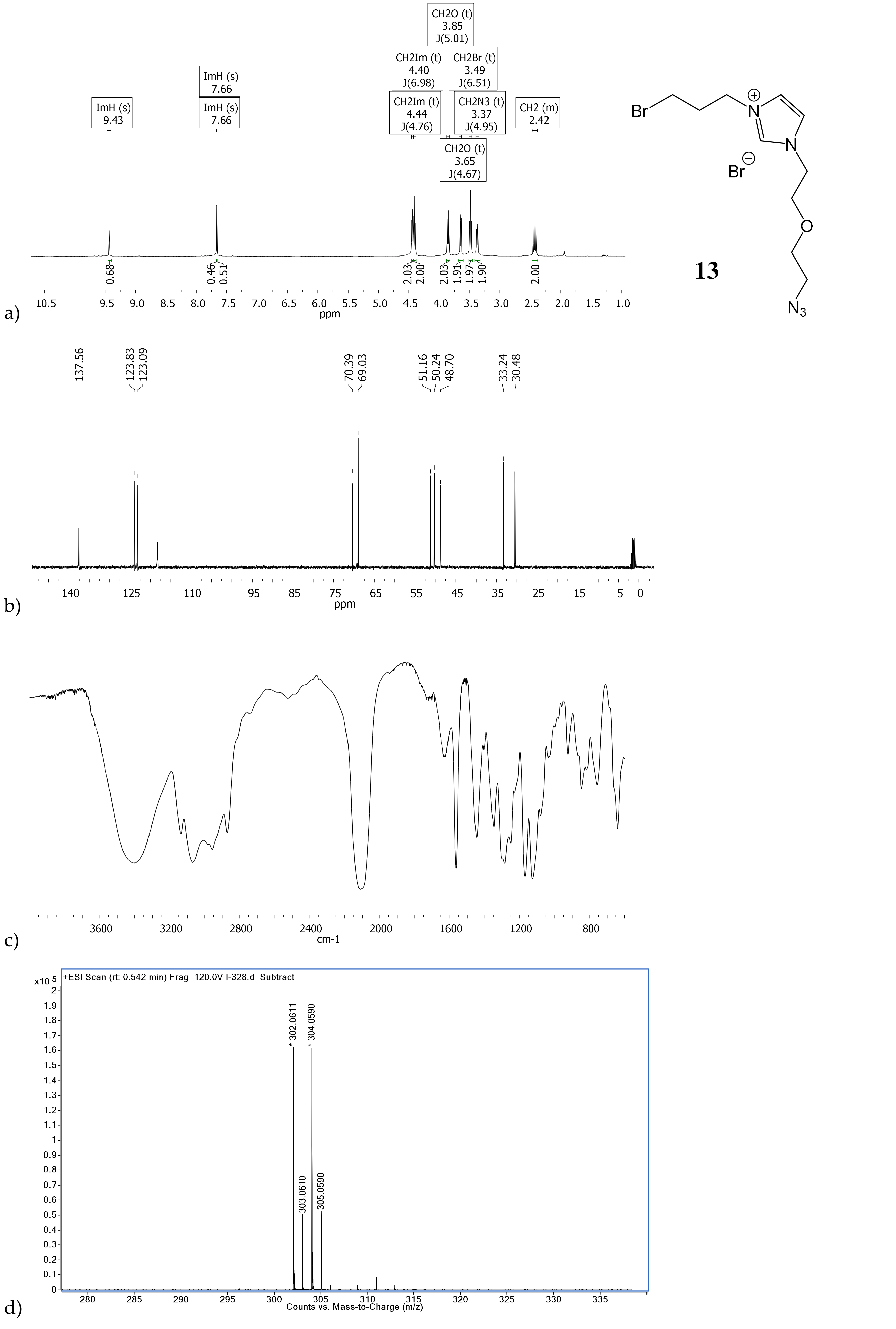

Figure S4. NMR ^{1}H (a), $^{13}C\{^{1}H\}$ (b), FTIR (c), and ESI (d) spectra of *3-(2-(2-azidoethoxy)ethyl)-1-(3-bromopropyl)-1H-imidazolium bromide* ***(13)***

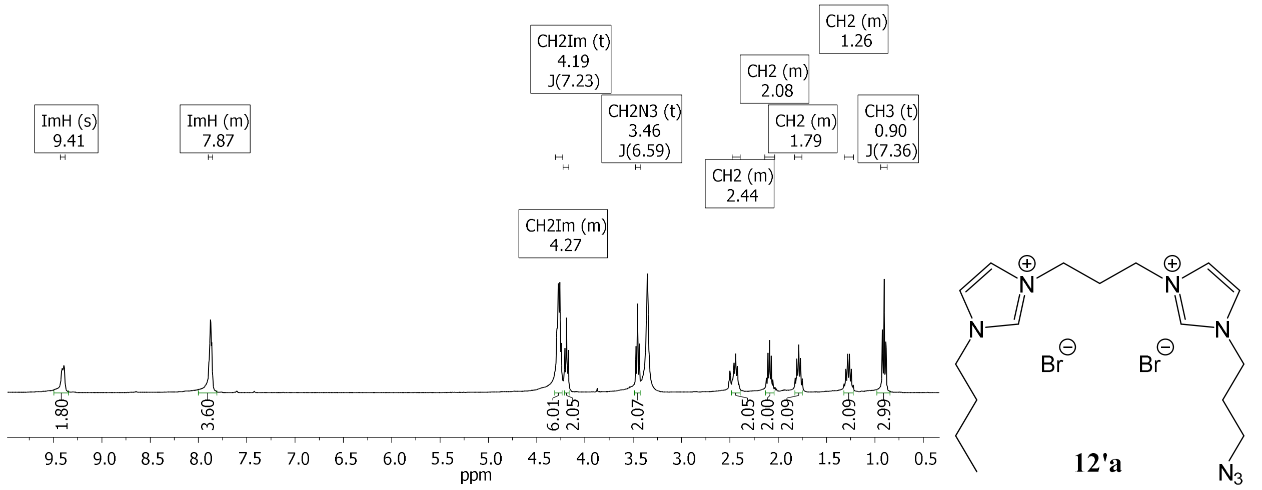

a)

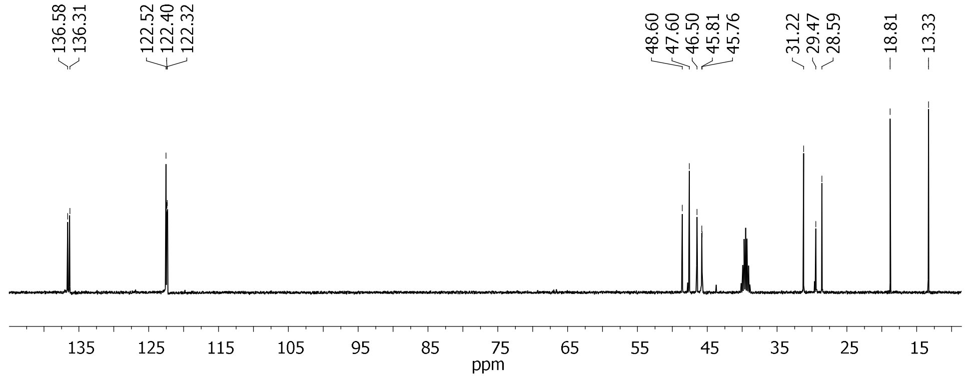

b)

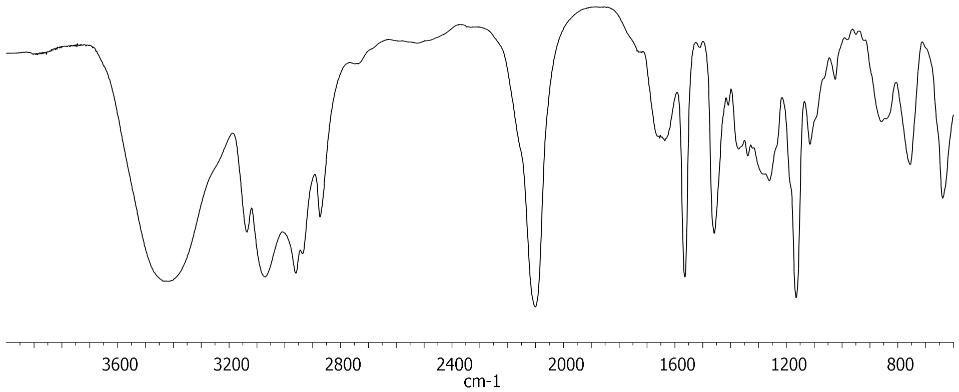

c)

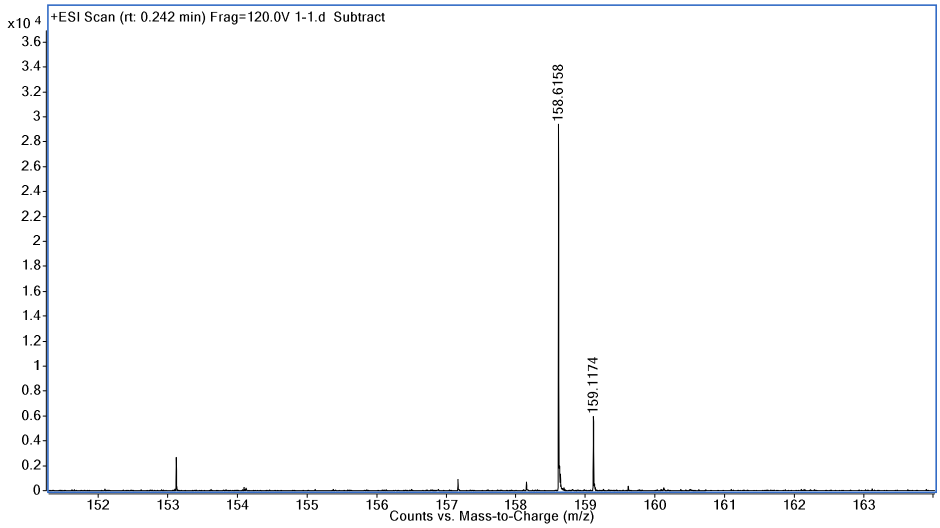

d)

Figure S5. NMR ^{1}H (a), ^{13}C{^{1}H} (b), FTIR (c), and ESI (d) spectra of *3-(3-azidopropyl)-1-(3-(1-butyl-1H-imidazol-3-yl)propyl)-1H-imidazolium dibromide (**12a**)*

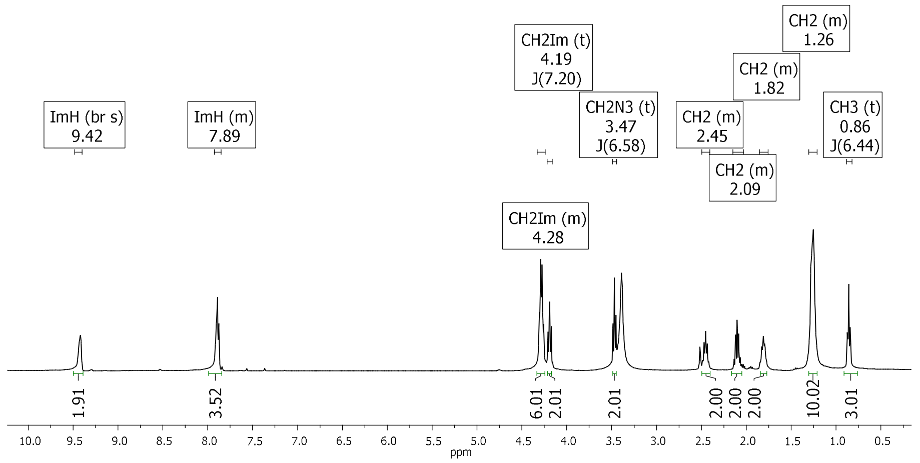

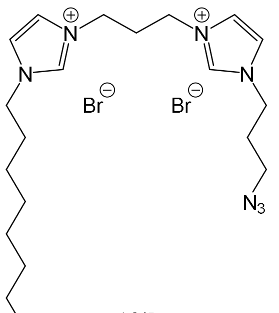

a)

12'b

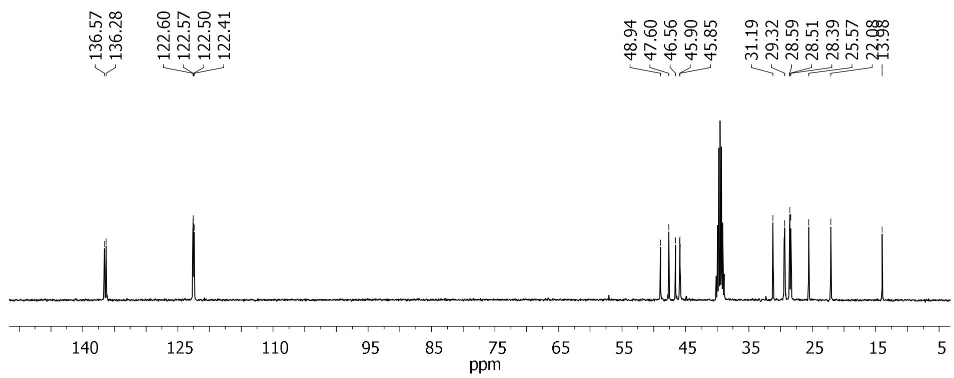

b)

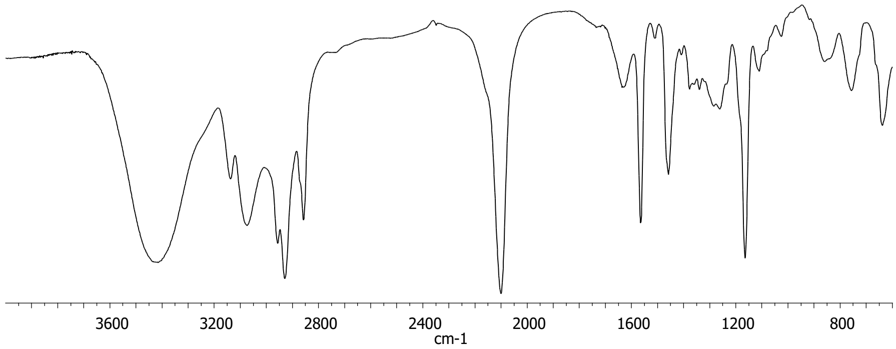

c)

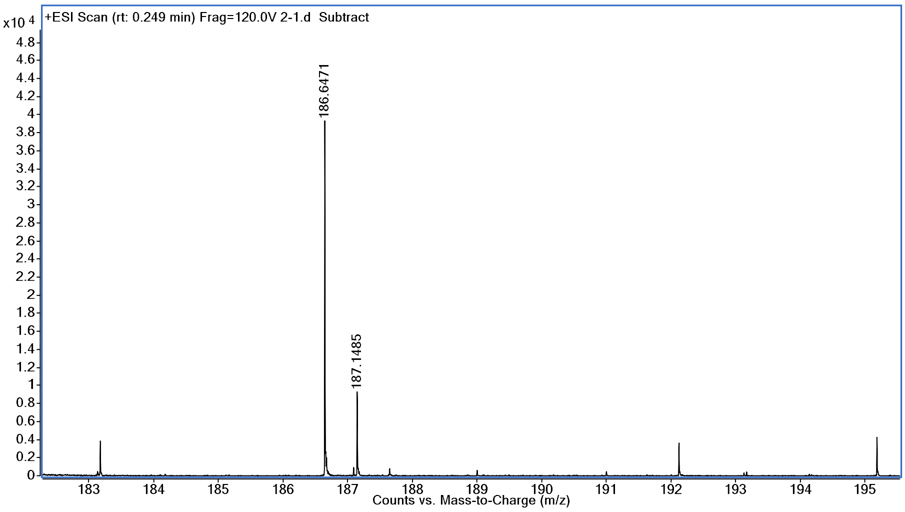

d)

Figure S6. NMR ^{1}H (a), $^{13}C\{^{1}H\}$ (b), FTIR (c), and ESI (d) spectra of *3-(3-azidopropyl)-1-(3-(1-octyl-1H-imidazol-3-yl)propyl)-1H-imidazolium dibromide (**12b**)*

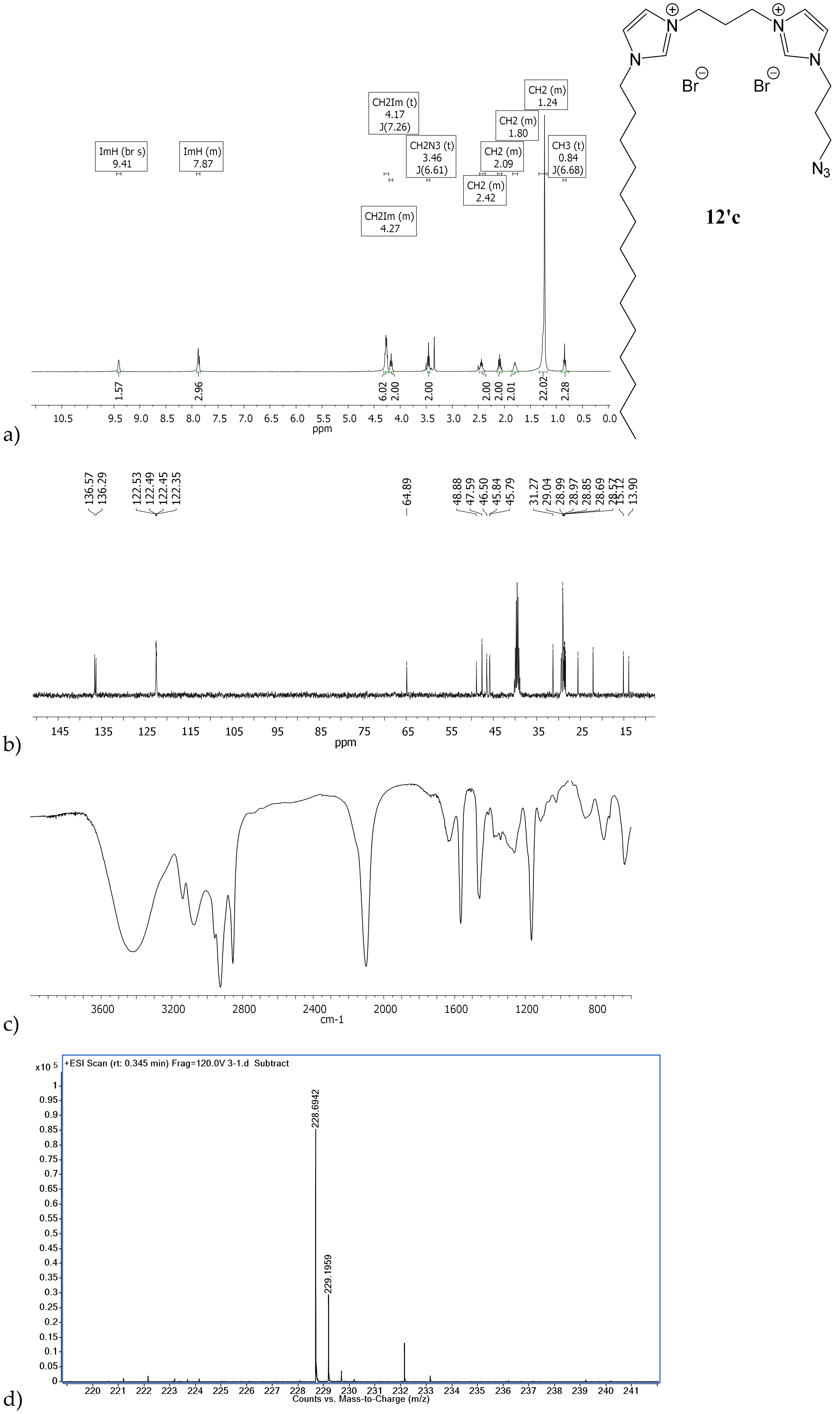

Figure S7. NMR ^{1}H (a), $^{13}C\{^{1}H\}$ (b), FTIR (c), and ESI (d) spectra of *3-(3-azidopropyl)-1-(3-(1-tetradecyl-1H-imidazol-3-yl)propyl)-1H-imidazolium dibromide (**12c**)*

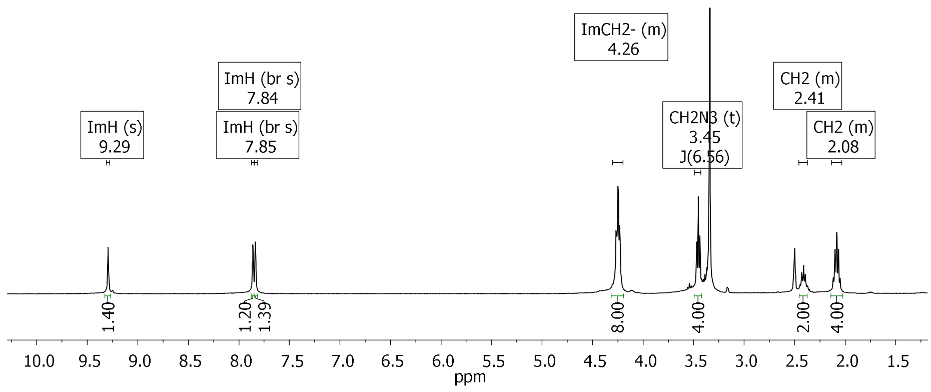

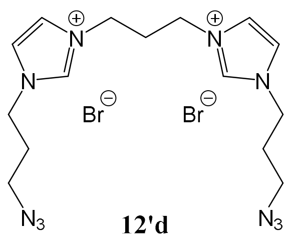

a) **12'd**

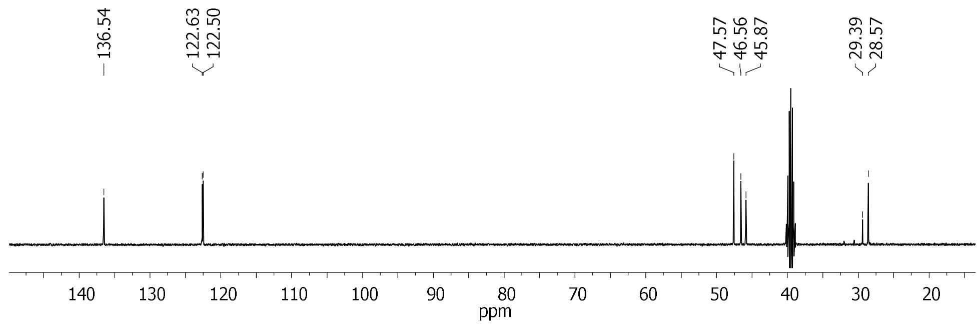

b)

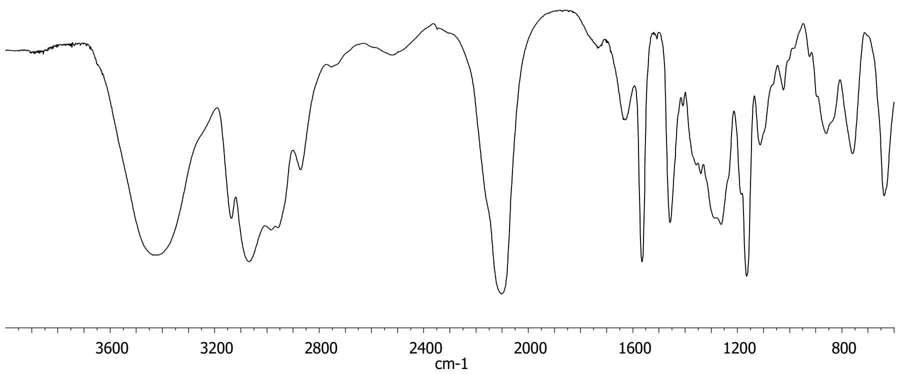

c)

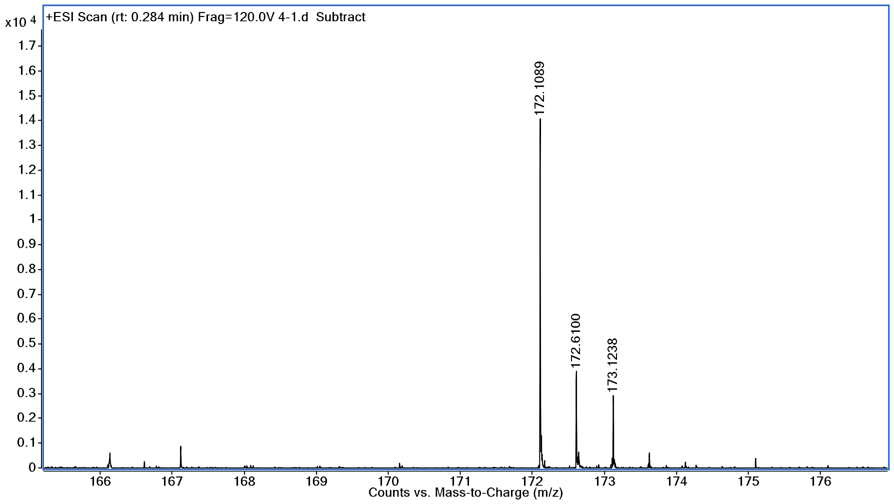

d)

Figure S8. NMR ^{1}H (a), $^{13}C\{^{1}H\}$ (b), FTIR (c), and ESI (d) spectra of *3-(3-azidopropyl)-1-(3-(1-tetradecyl-1H-imidazol-3-yl)propyl)-1H-imidazolium dibromide* (***12d***)

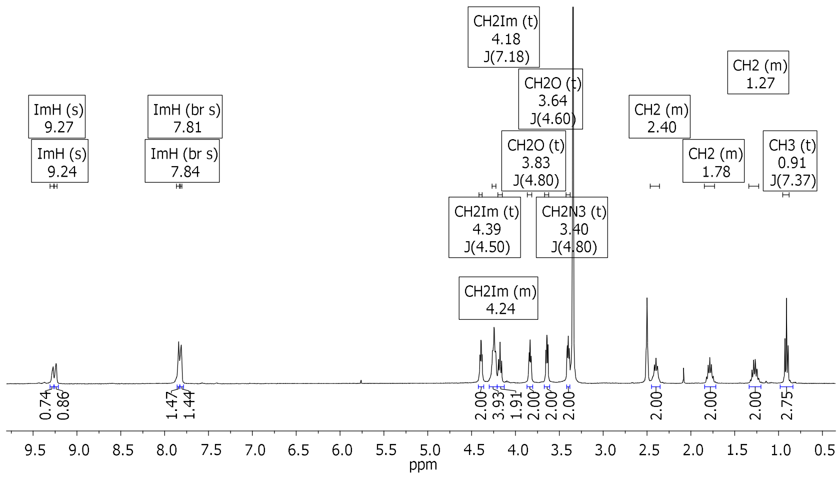

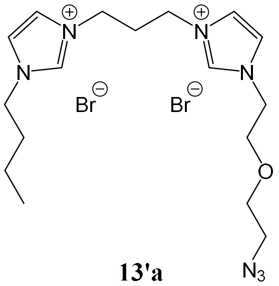

a)

13'a

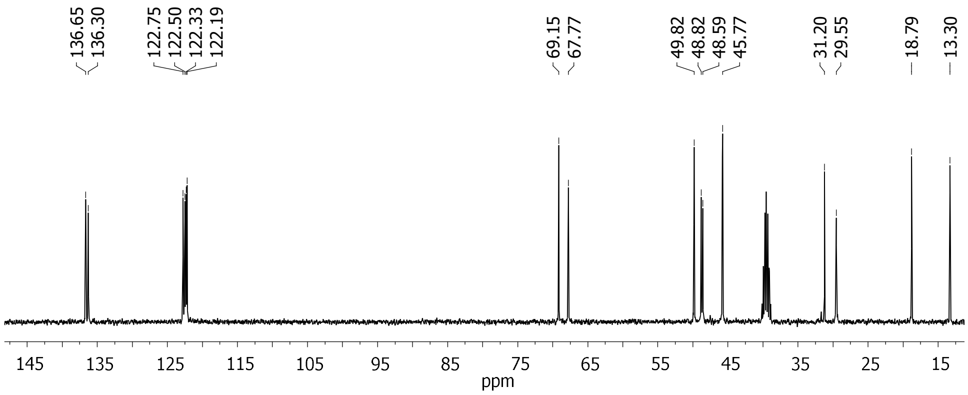

b)

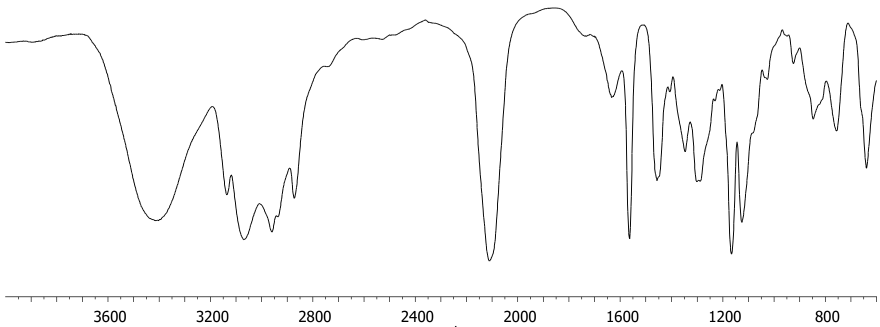

c)

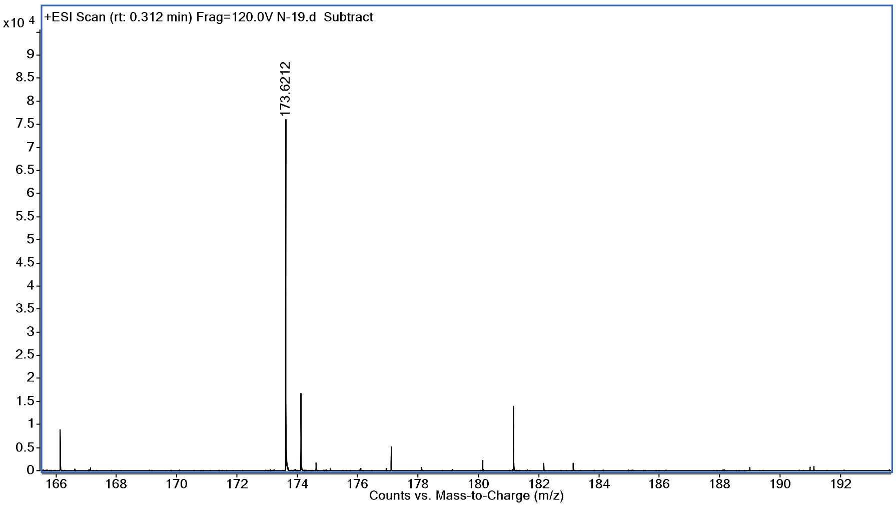

d)

Figure S9. NMR ^{1}H (a), $^{13}C\{^{1}H\}$ (b), FTIR (c), and ESI (d) spectra of *1-(2-(2-azidoethoxy)ethyl)-3-(3-(1-butyl-1H-imidazol-3-yl)propyl)-1H-imidazolium dibromide (**13a**)*

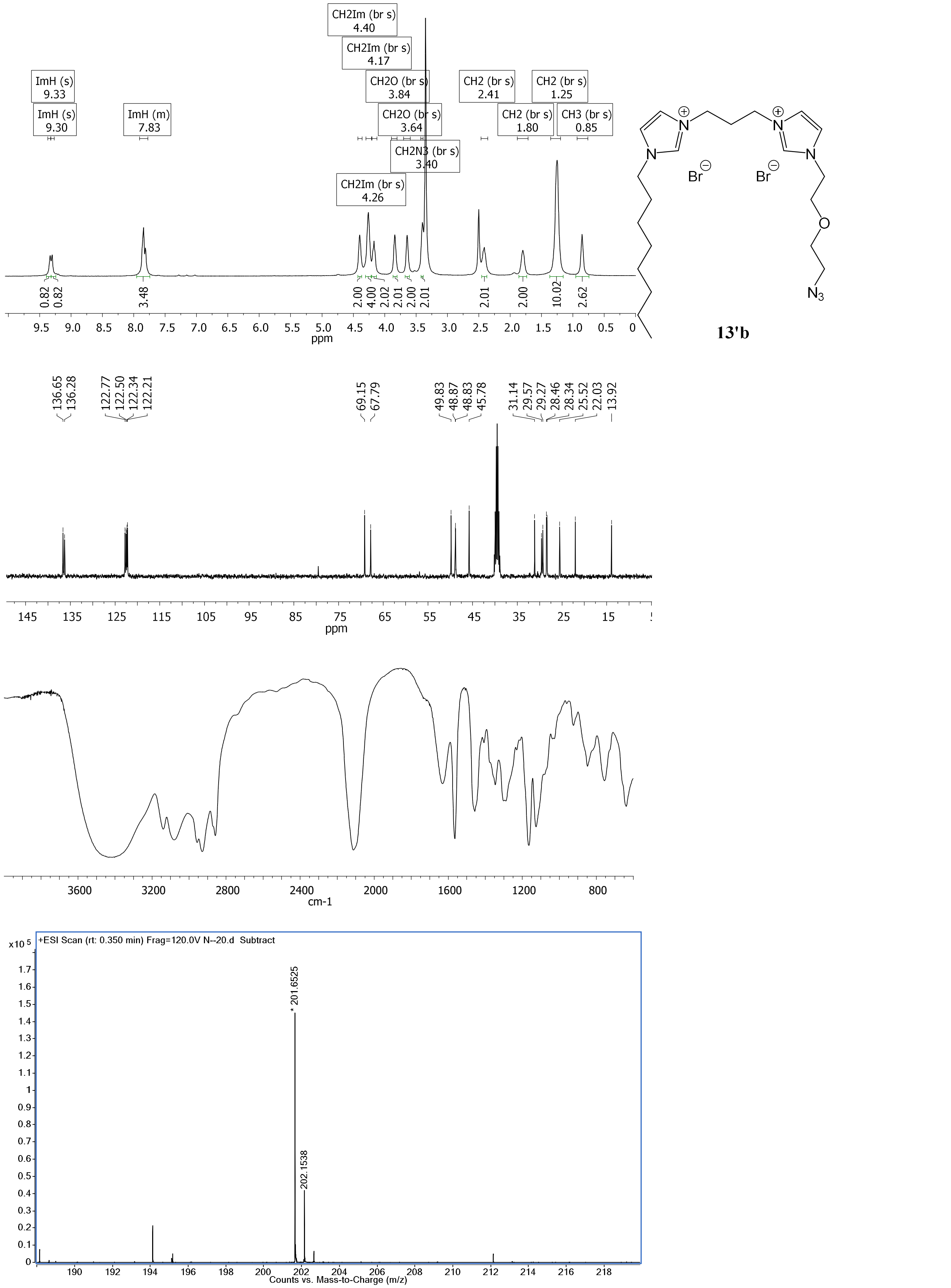

a)

b)

c)

d)

Figure S10. NMR ^1H (a), $^{13}C\{^1H\}$ (b), FTIR (c), and ESI (d) spectra of *1-(2-(2-azidoethoxy)ethyl)-3-(3-(1-octyl-1H-imidazol-3-yl)propyl)-1H-imidazolium dibromide (**13b**)*

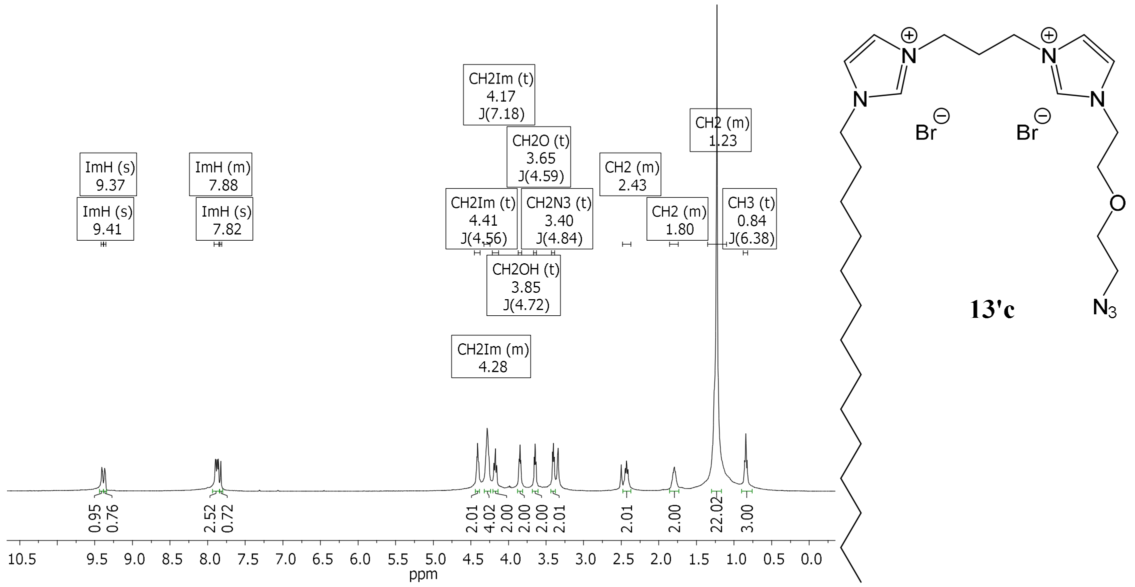

a)

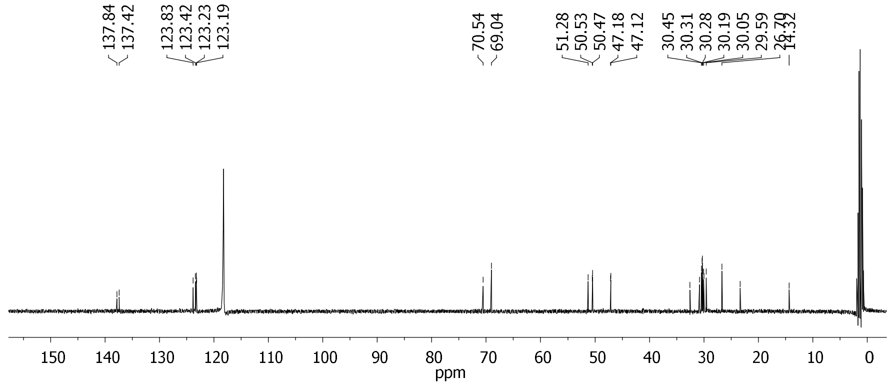

b)

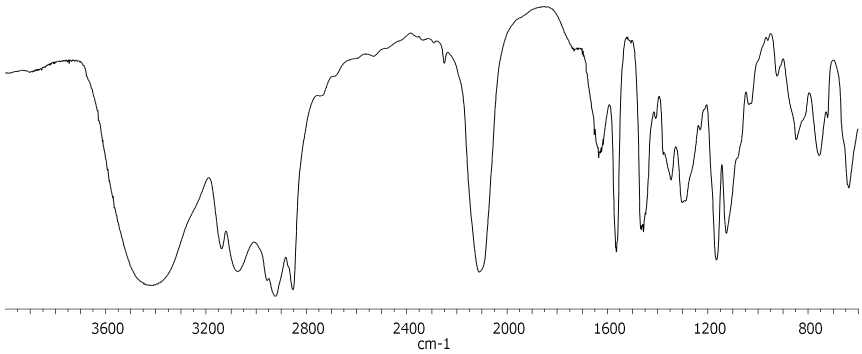

c)

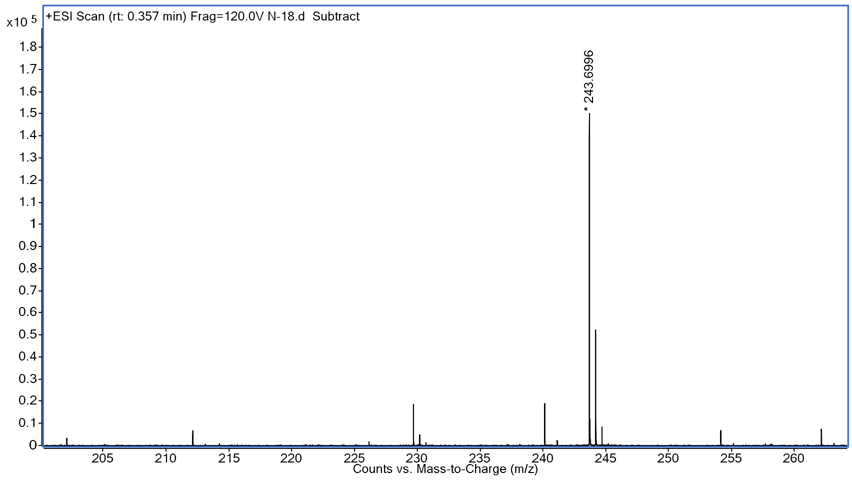

d)

Figure S11. NMR ^{1}H (a), ^{13}C{^{1}H} (b), FTIR (c), and ESI (d) spectra of *1-(2-(2-azidoethoxy)ethyl)-3-(3-(1-tetradecyl-1H-imidazol-3-yl)propyl)-1H-imidazolium dibromide* (**13c**)

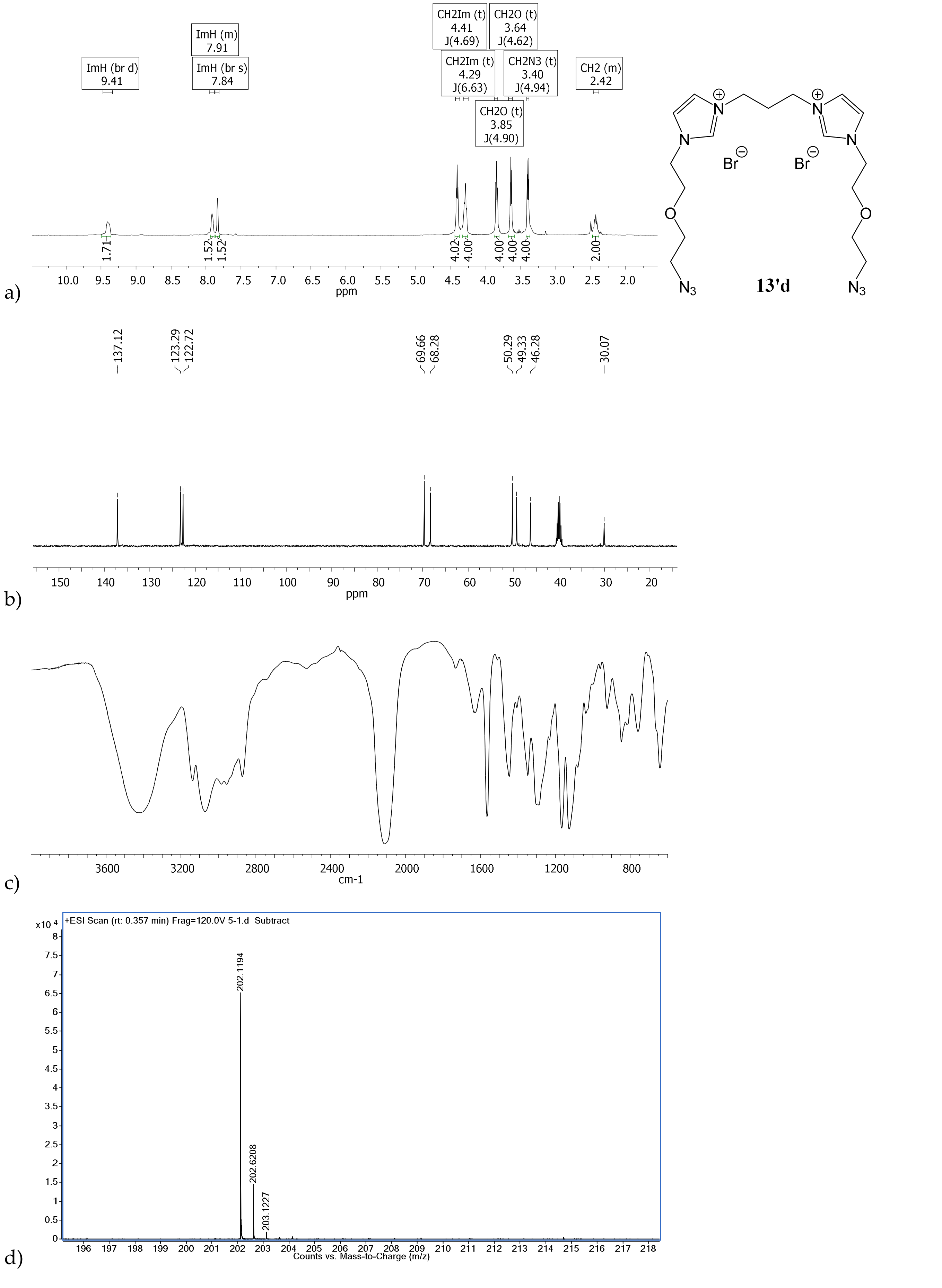

Figure S12. NMR ^{1}H (a), ^{13}C{^{1}H} (b), FTIR (c), and ESI (d) spectra of *3,3′-(propane-1,3-diyl)bis(1-(2-(2-azidoethoxy)ethyl)-1H-imidazol-3-ium) dibromide (**13d**)*

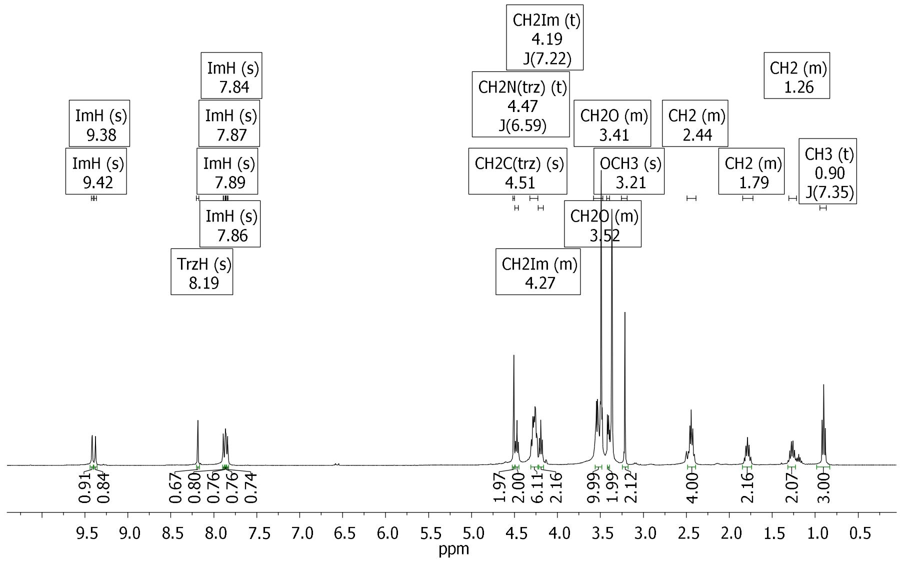

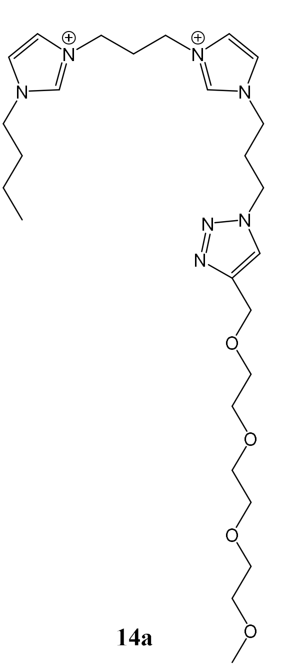

14a

a)

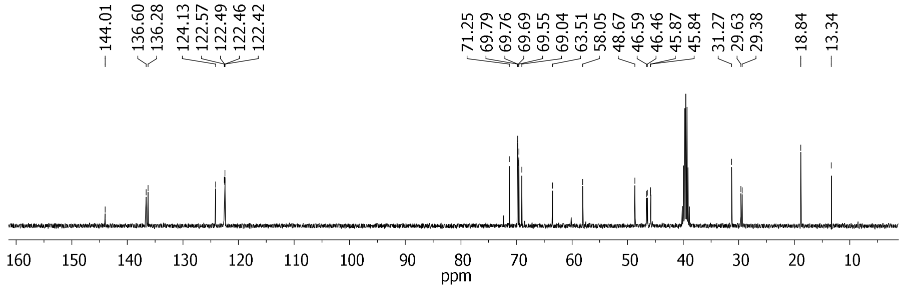

b)

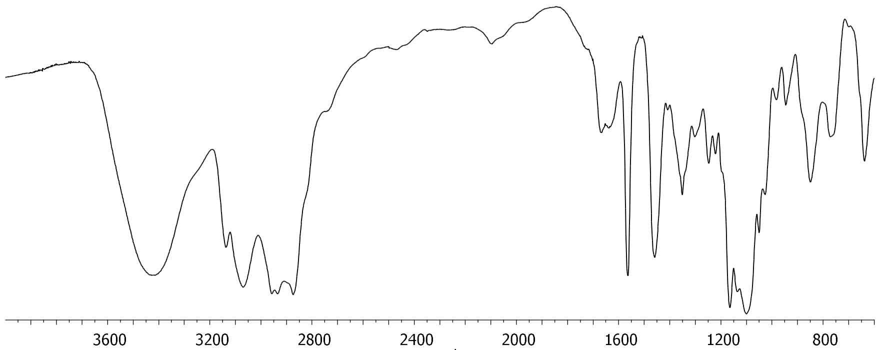

c)

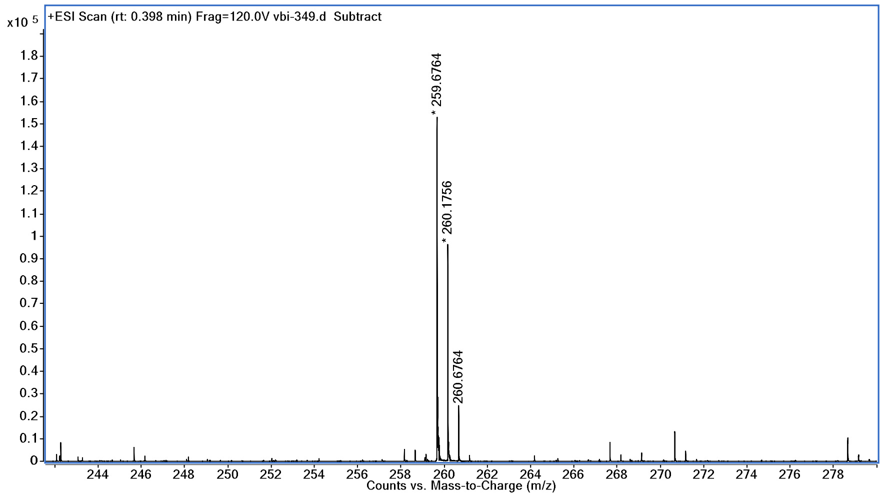

d)

Figure S13. NMR ^{1}H (a), $^{13}C\{^{1}H\}$ (b), FTIR (c), and ESI (d) spectra of *1-(3-(4-(2,5,8,11-tetraoxadodecyl)-1H-1,2,3-triazol-1-yl)propyl)-3-(3-(1-butyl-1H-imidazol-3-ium-3-yl)propyl)-1H-imidazol-3-ium dibromide* (***14a***)

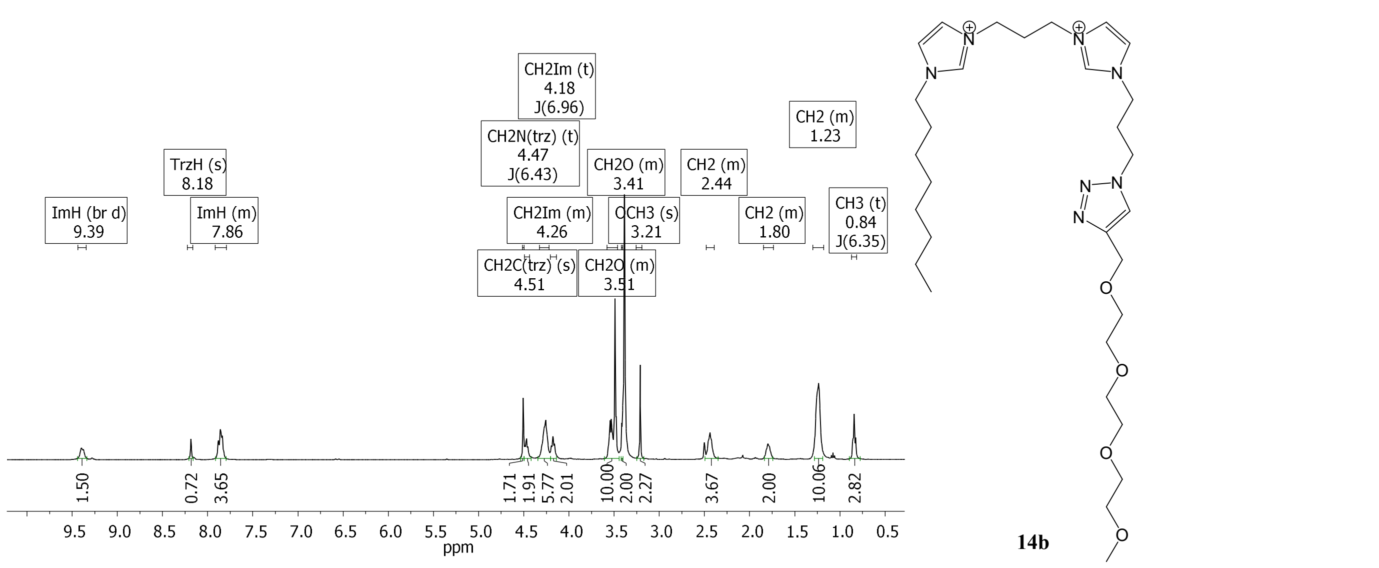

a)

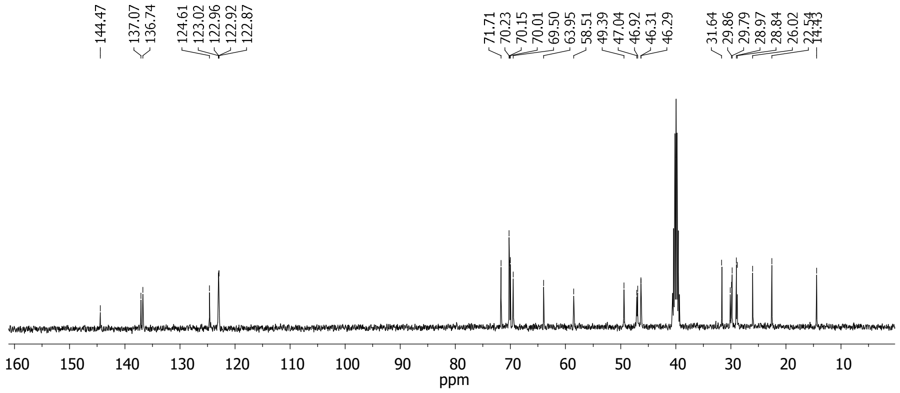

b)

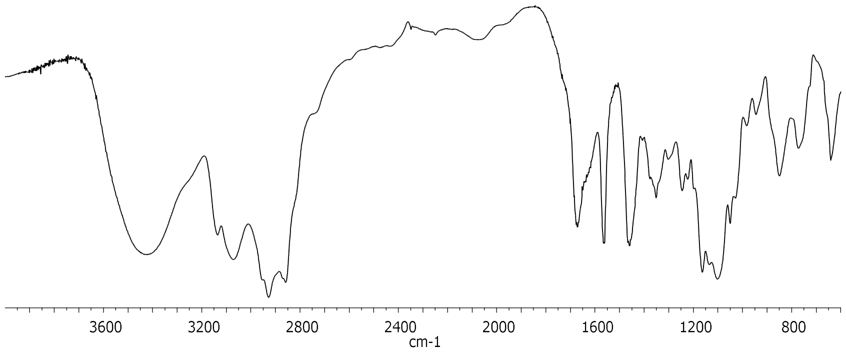

c)

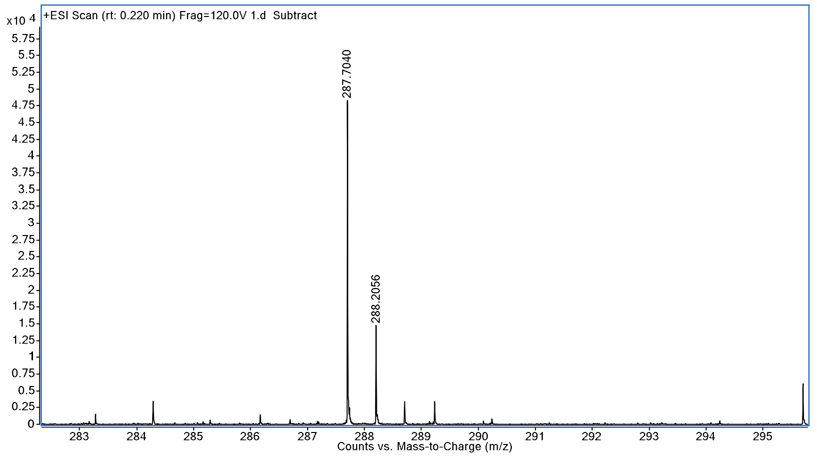

d)

Figure S14. NMR ^{1}H (a), ^{13}C{^{1}H} (b), FTIR (c), and ESI (d) spectra of *1-(3-(4-(2,5,8,11-tetraoxadodecyl)-1H-1,2,3-triazol-1-yl)propyl)-3-(3-(1-octyl-1H-imidazol-3-ium-3-yl)propyl)-1H-imidazol-3-ium dibromide* (***14b***)

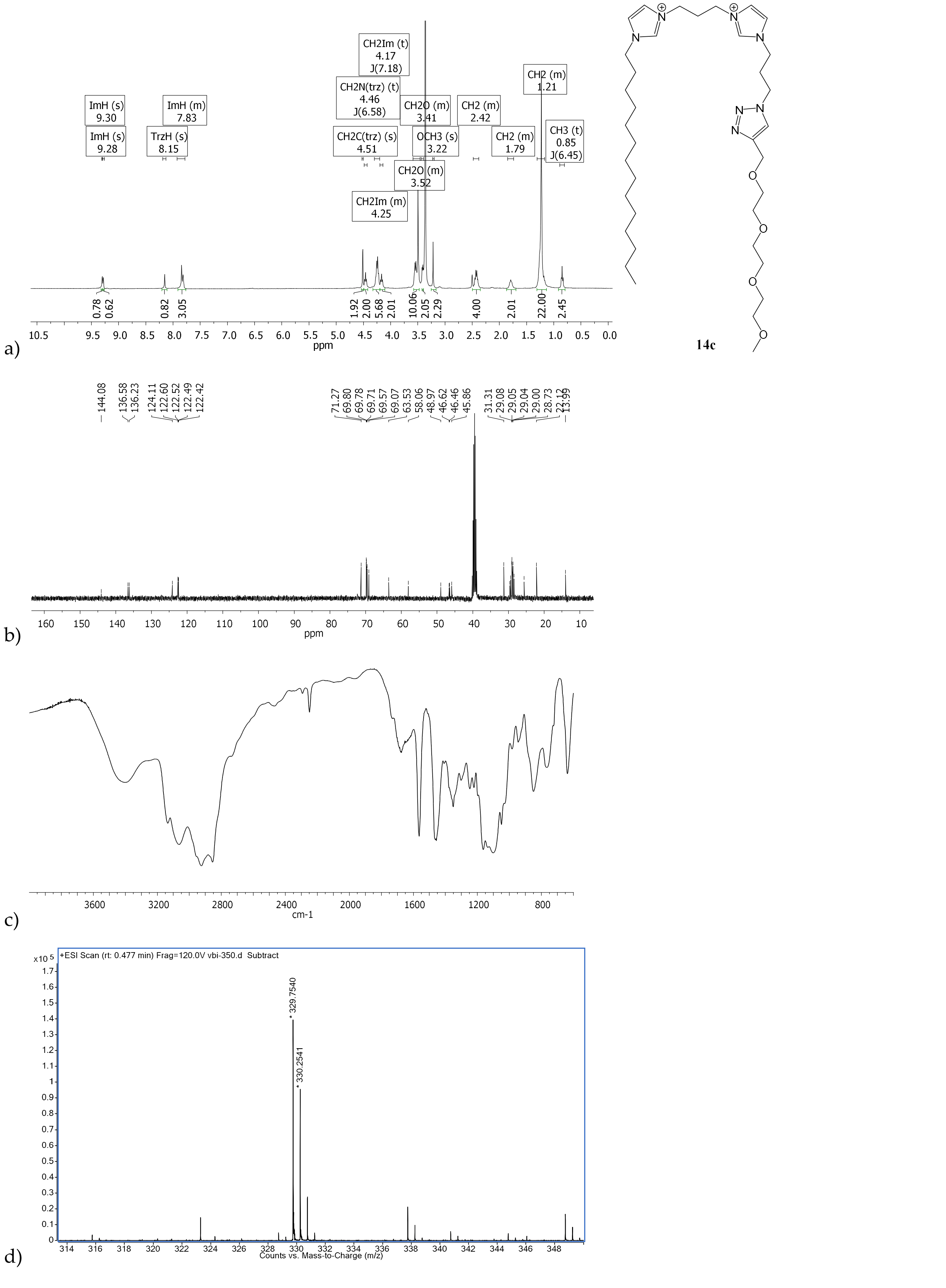

Figure S15. NMR ^{1}H (a), $^{13}C\{^{1}H\}$ (b), FTIR (c), and ESI (d) spectra of *1-(3-(4-(2,5,8,11-tetraoxadodecyl)-1H-1,2,3-triazol-1-yl)propyl)-3-(3-(1-tetradecyl-1H-imidazol-3-ium-3-yl)propyl)-1H-imidazol-3-ium dibromide* (**14c**)

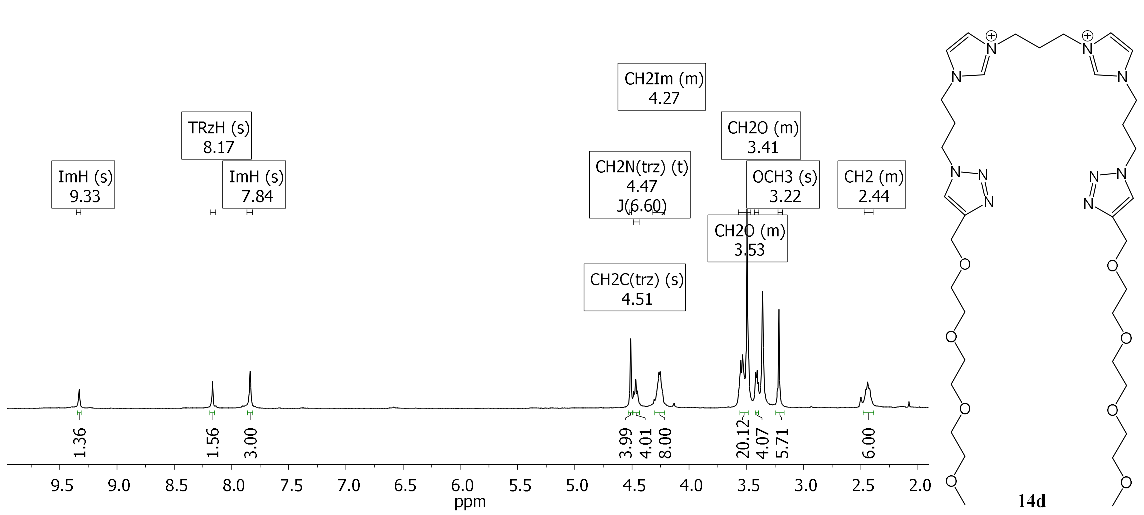

a)

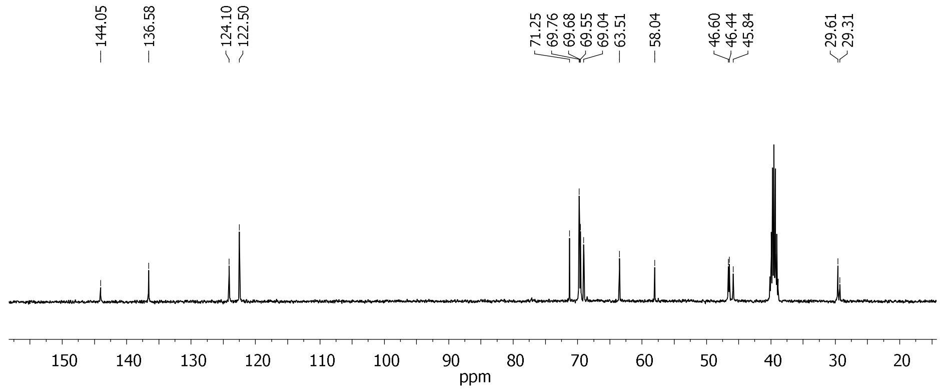

b)

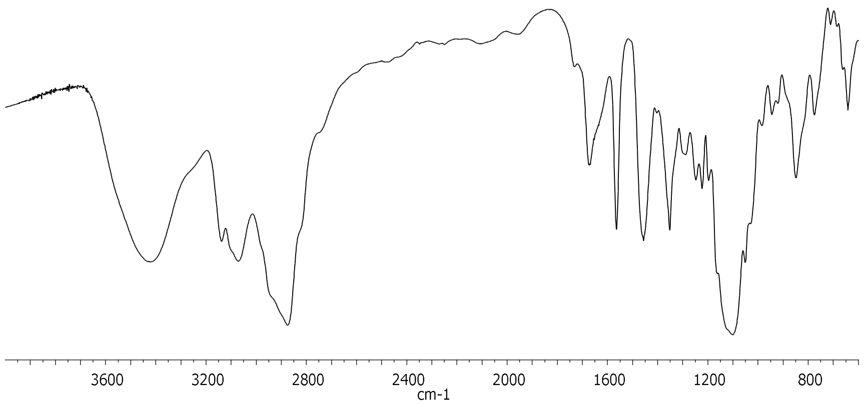

c)

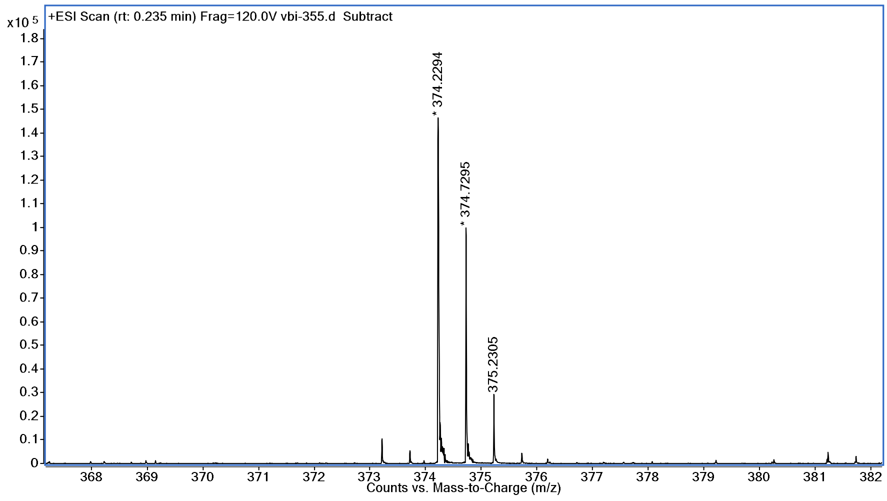

d)

Figure S16. NMR ^{1}H (a), ^{13}C{^{1}H} (b), FTIR (c), and ESI (d) spectra of *3,3′-(propane-1,3-diyl)bis(1-(3-(4-(2,5,8,11-tetraoxadodecyl)-1H-1,2,3-triazol-1-yl)propyl)-1H-imidazol-3-ium) dibromide* (***14d***)

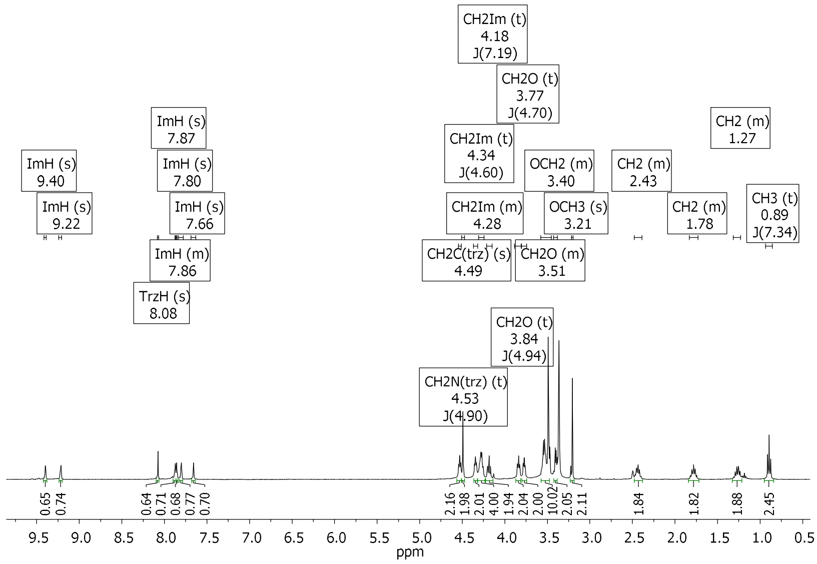

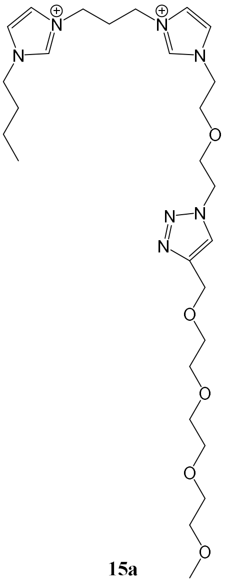

15a

a)

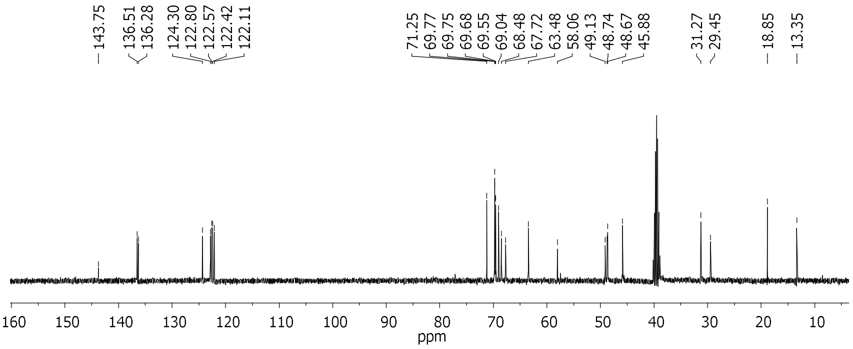

b)

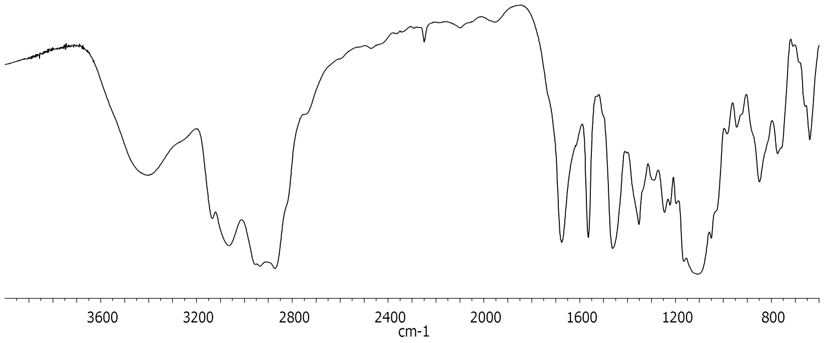

c)

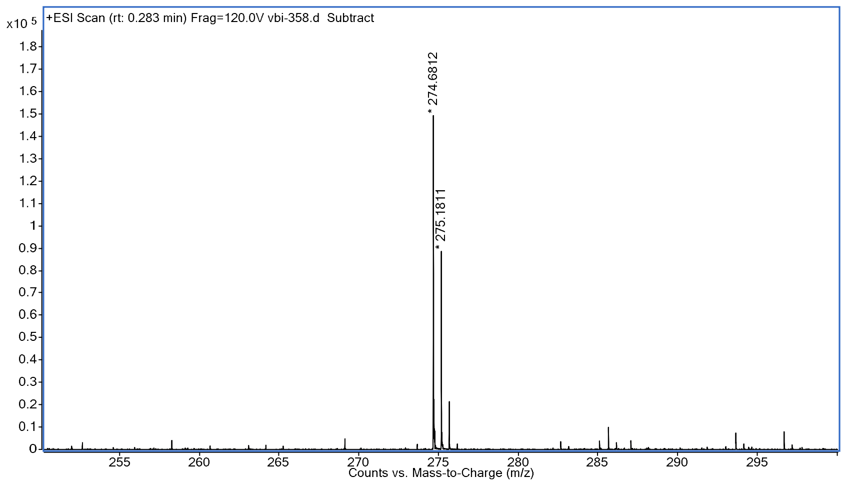

d)

Figure S17. NMR ^{1}H (a), ^{13}C{^{1}H} (b), FTIR (c), and ESI (d) spectra of *1-(2-(2-(4-(2,5,8,11-tetraoxadodecyl)-1H-1,2,3-triazol-1-yl)ethoxy)ethyl)-3-(3-(1-butyl-1H-imidazol-3-ium-3-yl)propyl)-1H-imidazol-3-ium dibromide* (***15a***)

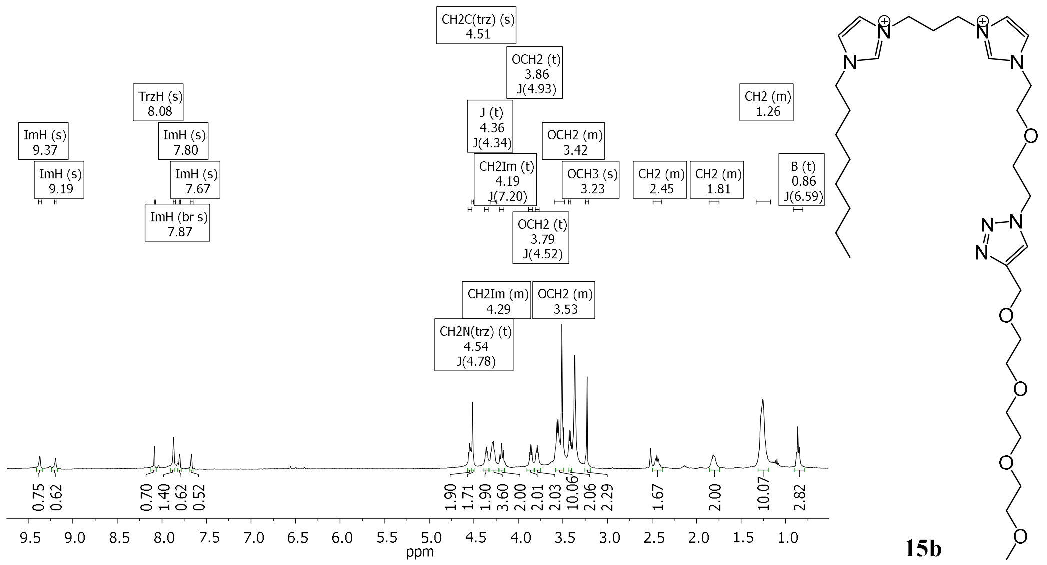

a)

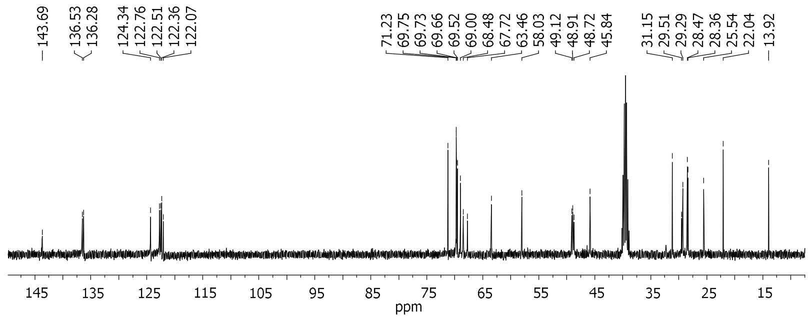

b)

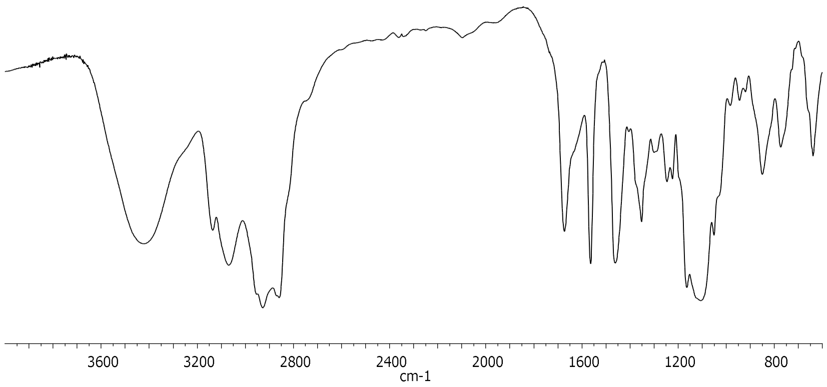

c)

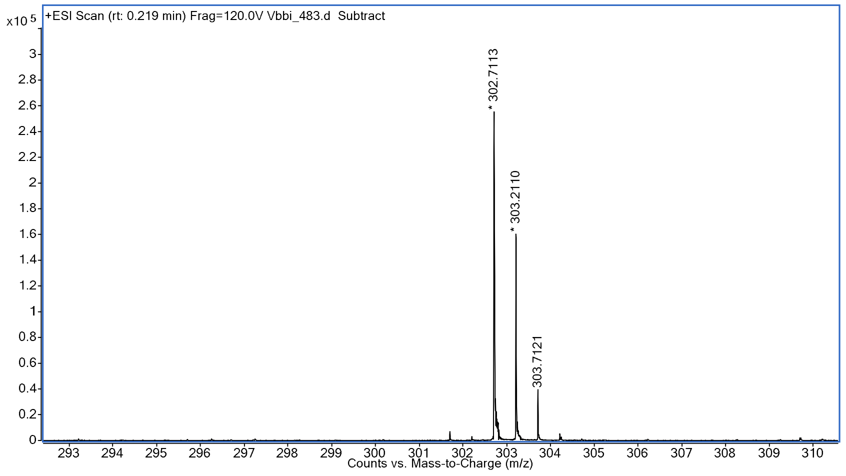

d)

Figure S18. NMR ^{1}H (a), $^{13}C\{^{1}H\}$ (b), FTIR (c), and ESI (d) spectra of *1-(2-(2-(4-(2,5,8,11-tetraoxadodecyl)-1H-1,2,3-triazol-1-yl)ethoxy)ethyl)-3-(3-(1-octyl-1H-imidazol-3-ium-3-yl)propyl)-1H-imidazol-3-ium dibromide (**15b**)*

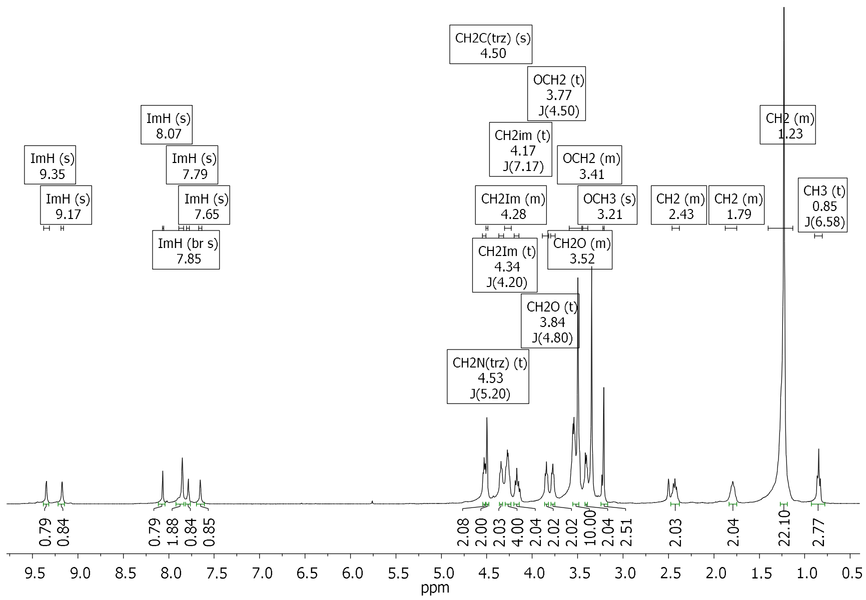

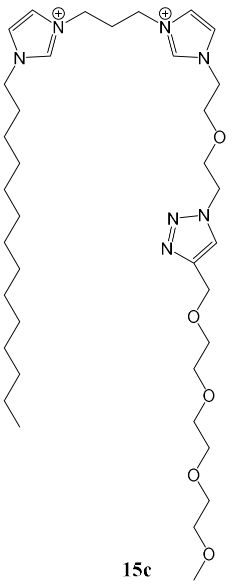

a)

15c

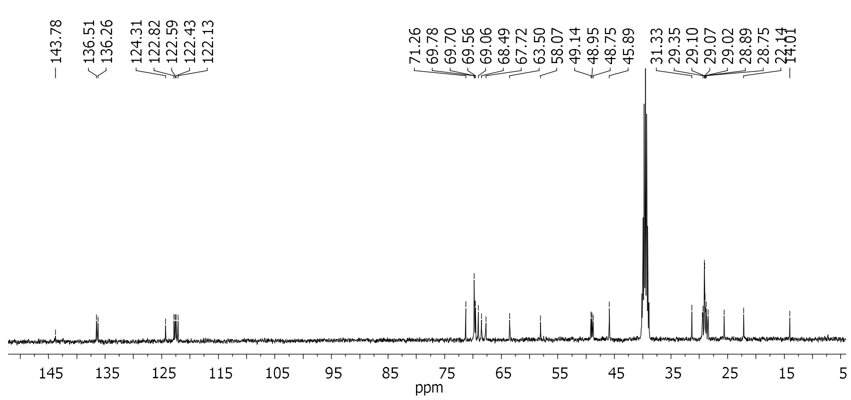

b)

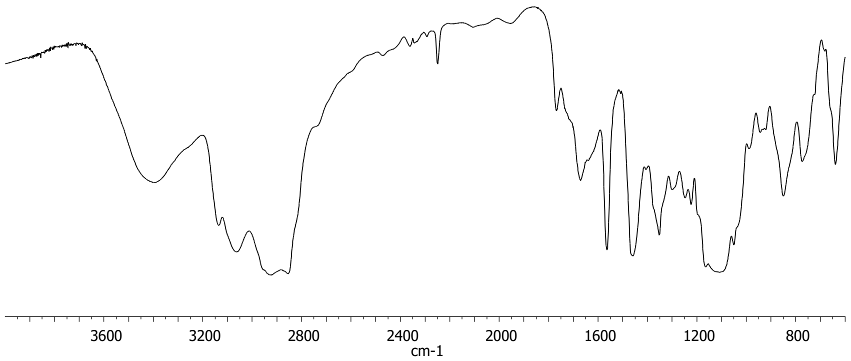

c)

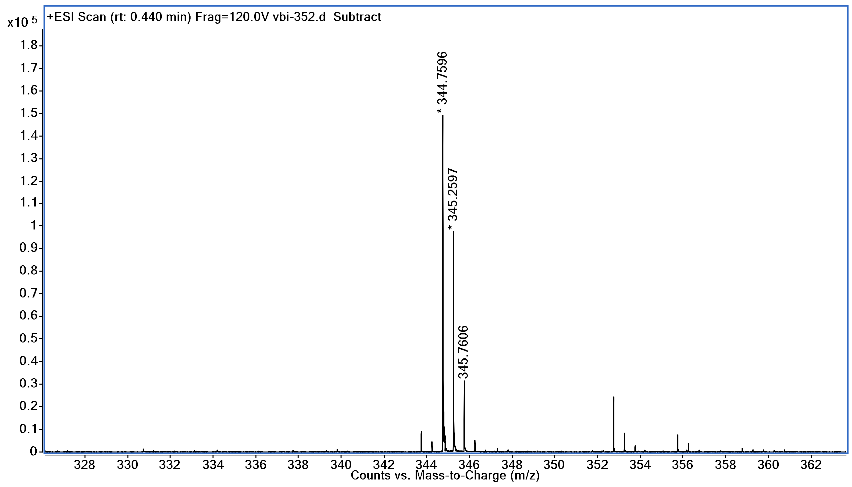

d)

Figure S19. NMR ^{1}H (a), ^{13}C{^{1}H} (b), FTIR (c), and ESI (d) spectra of *1-(2-(2-(4-(2,5,8,11-tetraoxadodecyl)-1H-1,2,3-triazol-1-yl)ethoxy)ethyl)-3-(3-(1-tetradecyl-1H-imidazol-3-ium-3-yl)propyl)-1H-imidazol-3-ium dibromide (**15c**)*

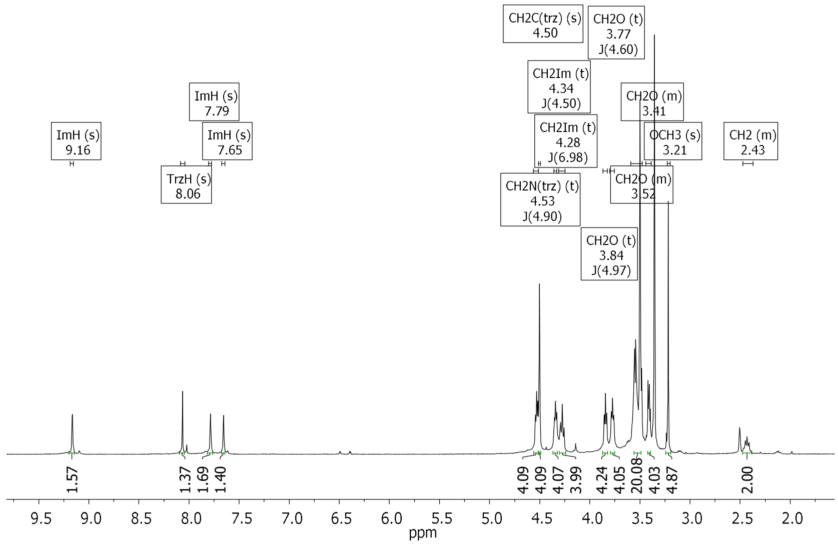

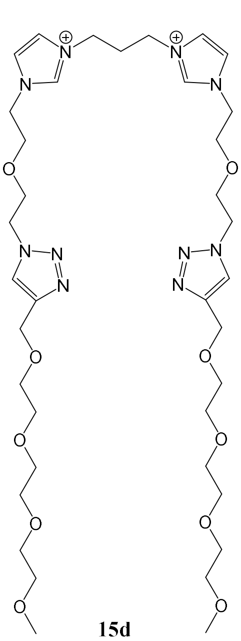

15d

a)

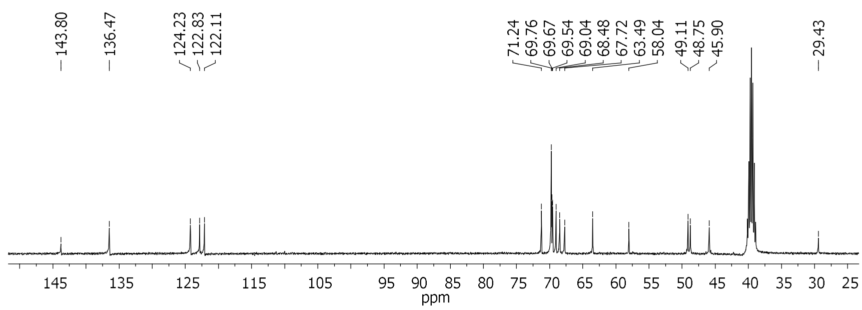

b)

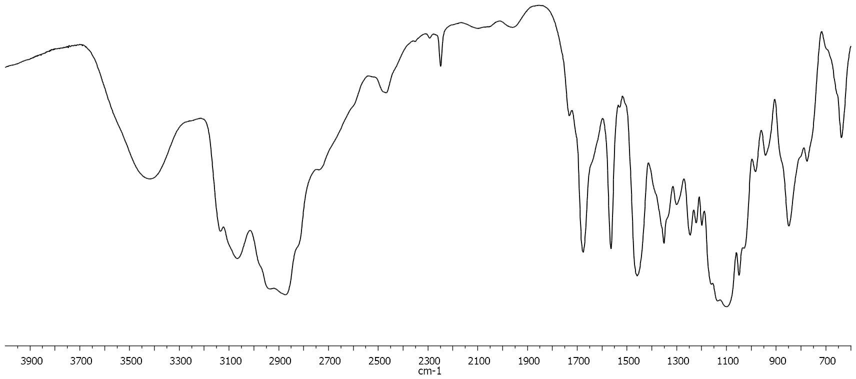

c)

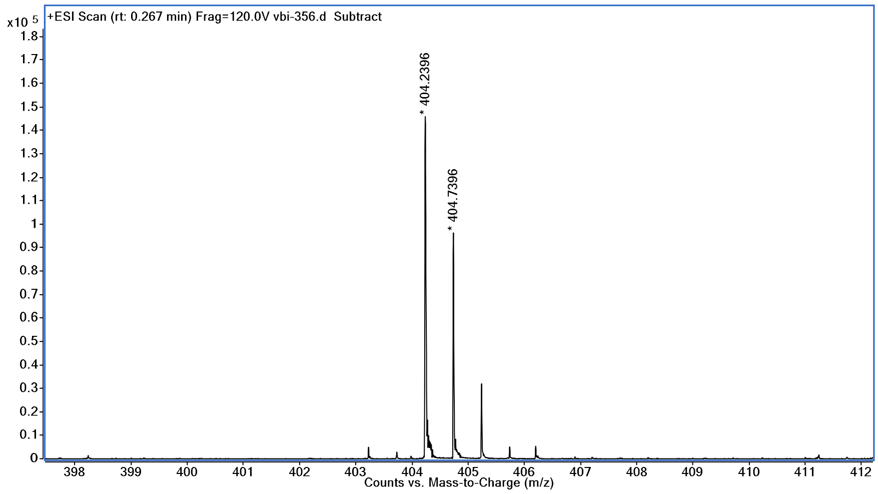

d)

Figure S20. NMR ^{1}H (a), $^{13}C\{^1H\}$ (b), FTIR (c), and ESI (d) spectra of *3,3′-(propane-1,3-diyl)bis(1-(2-(2-(4-(2,5,8,11-tetraoxadodecyl)-1H-1,2,3-triazol-1-yl)ethoxy)ethyl)-1H-imidazol-3-ium) dibromide* (***15d***)

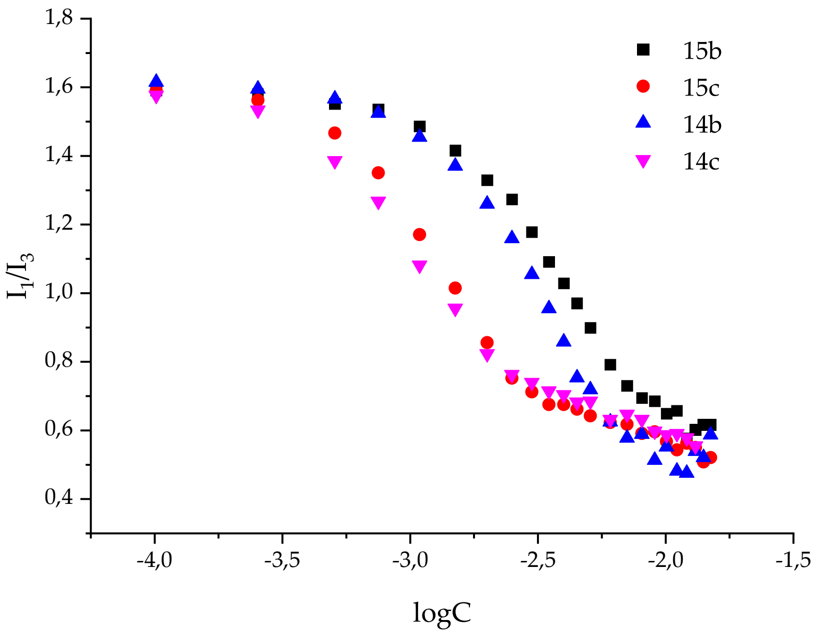

Figure S21. The ratio of the fluorescence intensities of the first (372 nm) and third (385 nm) pyrene emission peaks as a function of the concentration of amphiphilic molecules for binary surfactant/pyrene, [pyrene]= 1 μM

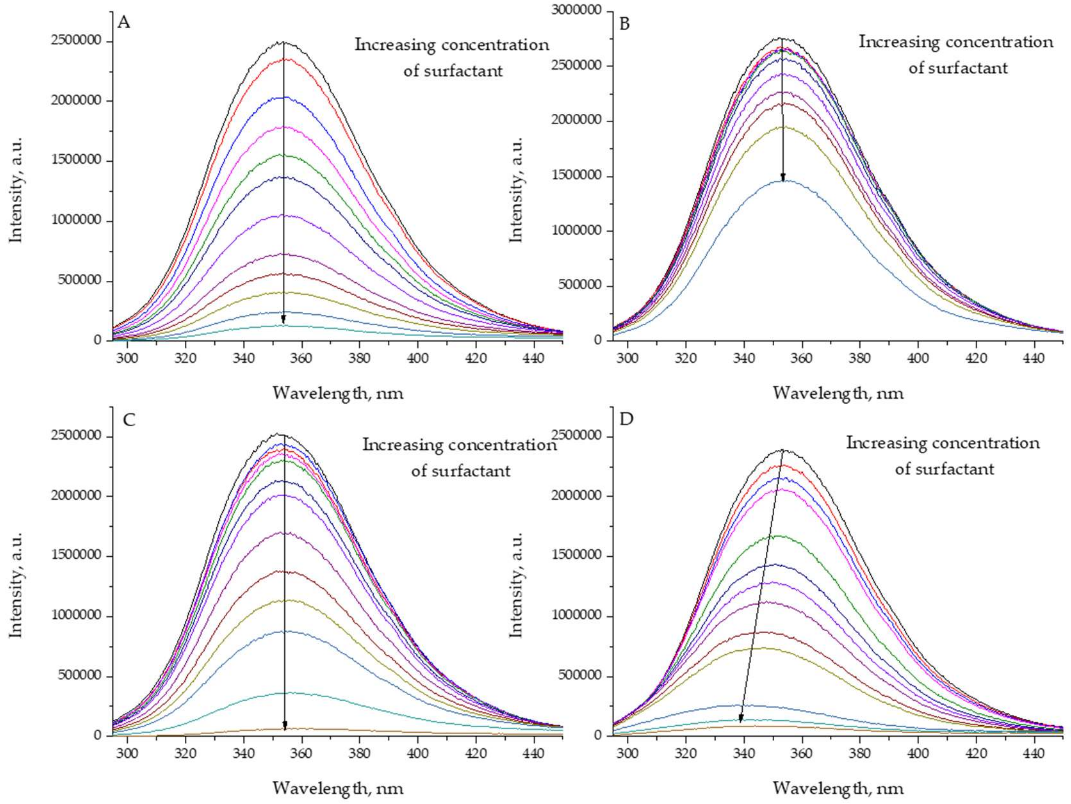

Figure S22. Emission spectra of BSA system in the absence and presence of various concentrations of **14d** (A), **14a** (B), **14b** (C), **14c** (D) in 25 mM Tris-HCl buffer solution with pH 7.4, [BSA] 10 μM.

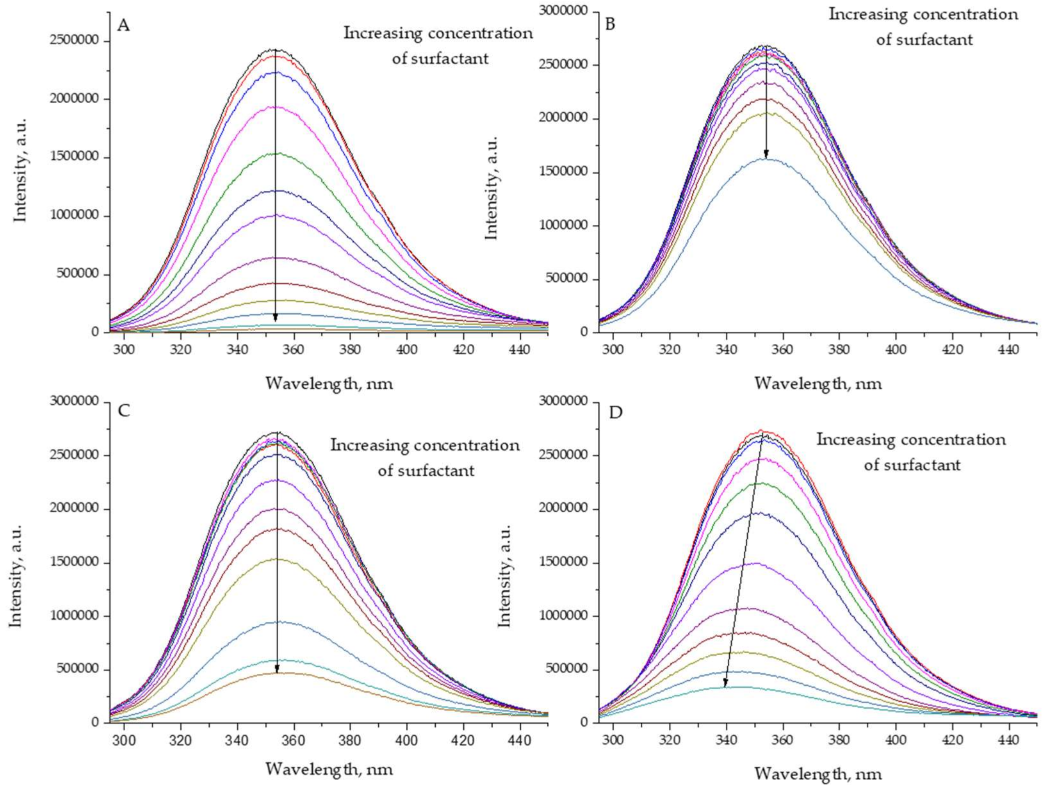

Figure S23. Emission spectra of BSA system in the absence and presence of various concentrations of **15d** (A), **15a** (B), **15b** (C), **15c** (D) in 25 mM Tris-HCl buffer solution with pH 7.4, [BSA] 10 μM.

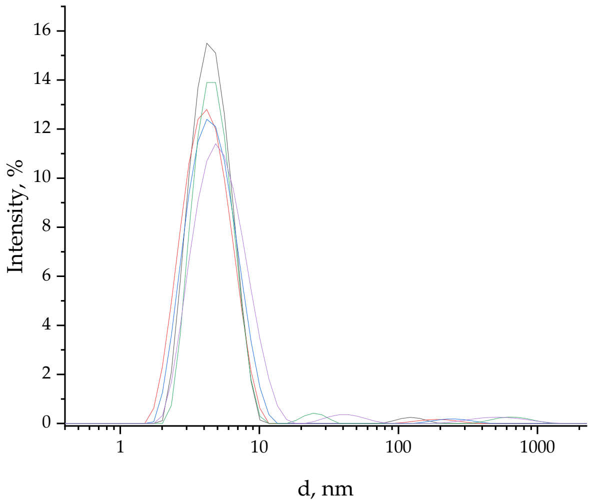

Figure S24. Dynamic light scattering particle size distribution of BSA (10 μM) suspended in 25 mM Tris-HCl buffer solution with pH 7.4.

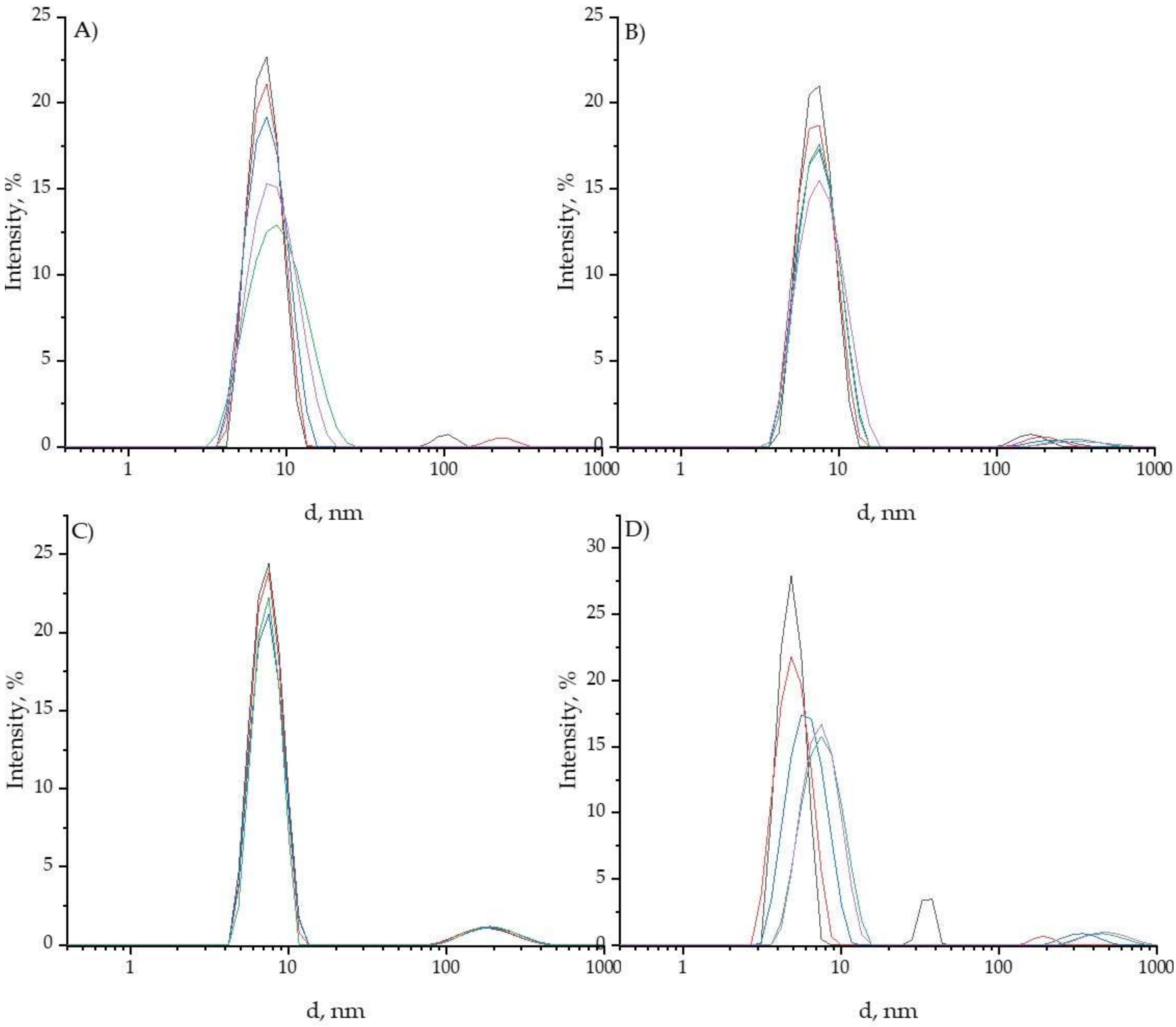

Figure S25. Dynamic light scattering particle size distribution of BSA (10 μM) in the presence of surfactant (700 μM): A) **14a**; B) **14b**; C) **14c**; D) **14d** in 25 mM Tris-HCl buffer solution with pH 7.4.

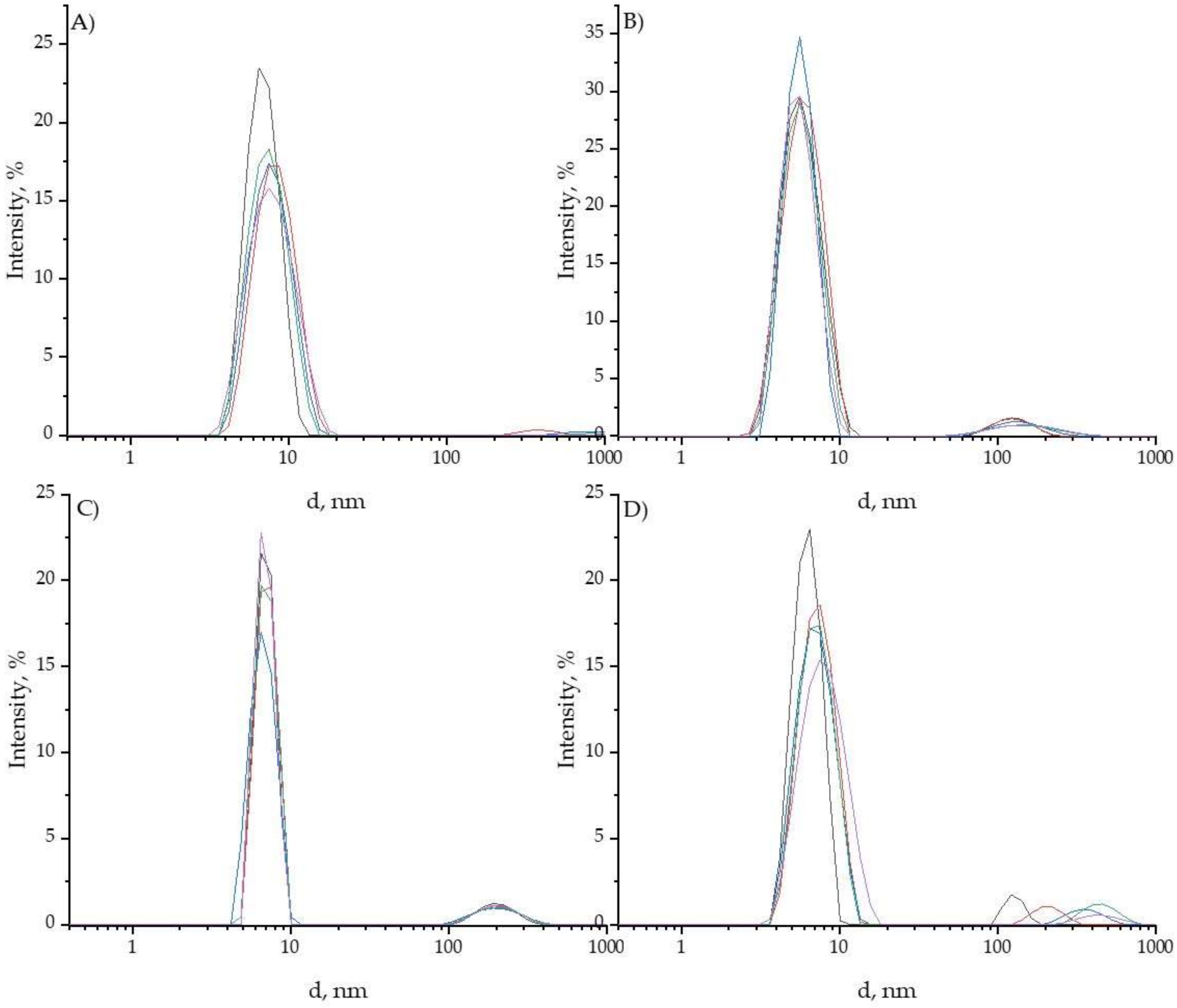

Figure S26. Dynamic light scattering particle size distribution of BSA (10 μM) in the presence of surfactant (700 μM): A) **15a**; B) **15b**; C) **15c**; D) **15d** in 25 mM Tris-HCl buffer solution with pH 7.4.
